# A Rag GTPase dimer code defines the regulation of mTORC1 by amino acids

**DOI:** 10.1038/s41556-022-00976-y

**Published:** 2022-09-12

**Authors:** Peter Gollwitzer, Nina Grützmacher, Sabine Wilhelm, Daniel Kümmel, Constantinos Demetriades

**Affiliations:** 1 Max Planck Institute for Biology of Ageing (MPI-AGE), Cologne, Germany; 2 Institute of Biochemistry, University of Münster, Münster, Germany; 3 Cologne Excellence Cluster on Cellular Stress Responses in Aging-Associated Diseases (CECAD), University of Cologne, Cologne, Germany

**Keywords:** TOR signalling, Lysosomes, Nutrient signalling, Molecular modelling

## Abstract

Amino acid availability controls mTORC1 activity via a heterodimeric Rag GTPase complex that functions as a scaffold at the lysosomal surface, bringing together mTORC1 with its activators and effectors. Mammalian cells express four Rag proteins (RagA–D) that form dimers composed of RagA/B bound to RagC/D. Traditionally, the Rag paralogue pairs (RagA/B and RagC/D) are referred to as functionally redundant, with the four dimer combinations used interchangeably in most studies. Here, by using genetically modified cell lines that express single Rag heterodimers, we uncover a Rag dimer code that determines how amino acids regulate mTORC1. First, RagC/D differentially define the substrate specificity downstream of mTORC1, with RagD promoting phosphorylation of its lysosomal substrates TFEB/TFE3, while both Rags are involved in the phosphorylation of non-lysosomal substrates such as S6K. Mechanistically, RagD recruits mTORC1 more potently to lysosomes through increased affinity to the anchoring LAMTOR complex. Furthermore, RagA/B specify the signalling response to amino acid removal, with RagB-expressing cells maintaining lysosomal and active mTORC1 even upon starvation. Overall, our findings reveal key qualitative differences between Rag paralogues in the regulation of mTORC1, and underscore Rag gene duplication and diversification as a potentially impactful event in mammalian evolution.

## Abstract (web summary)

Gollwitzer, Grützmacher et al. and Figlia et al. establish that the various Rag GTPase genes and isoforms differentially regulate mTORC1 activity and distinctly modulate the responsiveness of mammalian cells to amino acid availability.

## Main

Nutrients are the building blocks for cells to grow and proliferate; hence, nutrient sensing mechanisms ensure that cells only grow when all necessary elements are available and conditions are optimal. The main nutrient sensor in cells is mechanistic target of rapamycin complex 1 (mTORC1), which is robustly regulated by amino acid (AA) availability^1–3^, and—directly or indirectly—controls virtually all homoeostatic processes, including cell growth, metabolism and secretion^4–9^.

A major site for mTORC1 activation is the lysosomal surface, where it is recruited by the heterodimeric Rag (ras-related GTP binding) GTPases, consisting—in mammalian cells—of RagA or RagB (‘small’ Rags) bound to RagC or RagD (‘large’ Rags). In AA sufficiency, an ‘active’ Rag dimer (containing GTP-bound RagA/B and GDP-bound RagC/D) recruits mTORC1 to lysosomes, where it is activated by another small GTPase, Rheb (ras homologue enriched in brain)^10–12^. In turn, active mTORC1 phosphorylates lysosomal (for example, transcription factor EB (TFEB) and transcription factor E3 (TFE3)) and non-lysosomal (for example, ribosomal protein S6 kinase (S6K)) substrates, to regulate various cellular processes such as lysosome biogenesis and protein synthesis^7,9^. In contrast, AA removal leads to inactivation of the Rag dimer and subsequent de-localization of mTORC1 away from lysosomes, which is part of its inactivation process^2,3,12,13^. Therefore, the Rags coordinate the cellular response to AA availability via the regulation of mTORC1 at the lysosomal surface.

Phenomena of gene duplication and divergence have driven evolution since the dawn of life and are generally considered a source of new protein functions^14,15^. Although paralogous genes usually code for proteins with similar structure and function, they often demonstrate specialized activities that contribute to the fine-tuning of key cellular processes^16–19^. Mammalian cells contain four Rag genes, designated *RRAGA*–*D*. With 90% AA sequence homology, the RagA and RagB proteins are very similar to each other^20^, which is also the case for RagC and RagD, which are ~80% identical^11,21,22^. Consequently, the different Rag dimers have so far been used interchangeably to study AA signalling to mTORC1. Moreover, despite the apparent sequence diversification between RagA and RagB, and between RagC and RagD, they are traditionally referred to as functionally redundant and equivalent to each other^23–26^. However, scattered observations in the literature hint at the existence of non-overlapping functions between the Rag paralogues. For instance, we have previously reported that tuberous sclerosis complex 2 (TSC2), a key negative regulator of Rheb and mTORC1, demonstrates strong preference for RagA binding over the other Rags^2^. Another example is leucyl-tRNA synthetase (LARS), an enzyme that binds and regulates RagD—but not RagC—in response to leucine supplementation^27,28^.

Driven by such observations, we hypothesized that the different Rags may be functionally divergent, and that the presence of two additional Rag paralogues in mammalian cells may be adding to the complexity of the regulation of mTORC1 by AAs. By using genetically modified cell lines that express only one of the four Rag dimer combinations, we now show that these are qualitatively different. We report two major dissimilarities: (1) whereas RagD-containing dimers are primarily responsible for the lysosomal recruitment and activation of mTORC1 (as seen by TFEB/TFE3 phosphorylation), both RagC and RagD can drive phosphorylation of its non-lysosomal targets (for example, S6K); (2) cells expressing RagA-containing dimers respond to AA withdrawal by robustly inactivating mTORC1, while RagB-containing dimers confer partial resistance to starvation. Furthermore, we provide a mechanistic explanation for the enhanced lysosomal tethering of RagD over RagC, characterize previously described, cancer-associated RagC mutants and identify regions in each Rag paralogue pair that are responsible for these functional differences.

## Results

### The RagC and RagD paralogues differentially regulate mTORC1

**Fig. 1:**
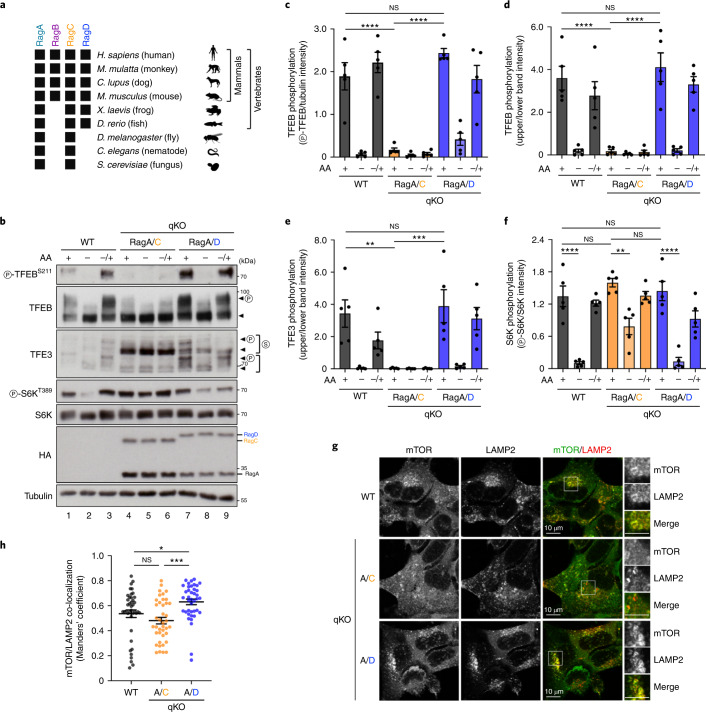
The RagC and RagD paralogues differentially regulate mTORC1. **a**, Schematic representation of the presence (black squares) or absence of the *RagA*–*D* genes in the genome of the indicated species. **b**, Immunoblots with lysates from HEK293FT WT, or qKO cells stably expressing HA-tagged RagA/C or RagA/D, treated with medium containing (+) or lacking (–) AA, in basal (+), starvation (–) or add-back (–/+) conditions, probed with the indicated antibodies. Arrowheads indicate bands corresponding to different protein forms, when multiple bands are present. P, phosphorylated form; S, SUMOylated form^60^. **c**–**f**, Quantification of TFEB (**c** and **d**), TFE3 (**e**) and S6K (**f**) phosphorylation from **b**. *n* = 5 independent experiments. **g**, Co-localization analysis of mTOR with LAMP2 (lysosomal marker) in HEK293FT WT, or qKO cells stably expressing RagA/C or RagA/D, using confocal microscopy. Magnified insets shown to the right. Scale bars, 10 μm. **h**, Quantification of mTOR/LAMP2 co-localization from *n* = 40 individual cells per condition from a representative experiment out of two independent replicates. Data in graphs shown as mean ± s.e.m. *P < 0.05, **P < 0.01, ***P < 0.005, ****P < 0.001. Source numerical data and unprocessed blots are available in source data. Source data

**Extended Data Fig. 1:**
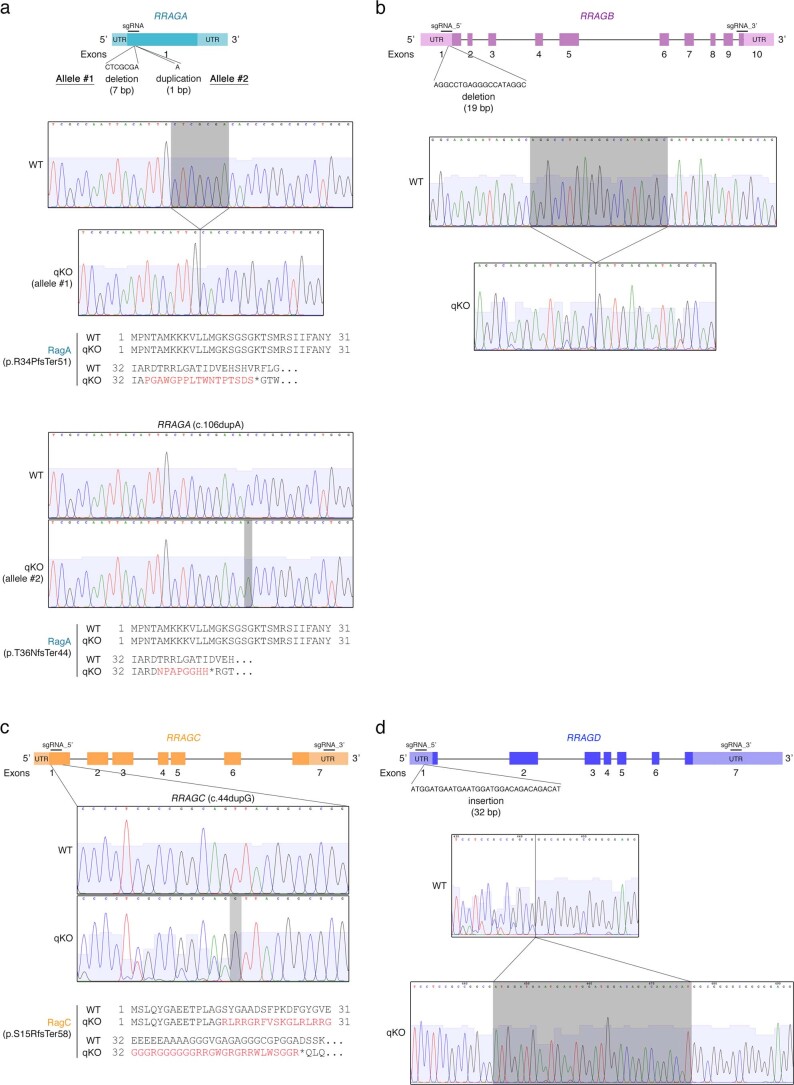
Characterization of the *Rag* genomic alterations in qKO HEK293FT cells. **(a)** CRISPR/Cas9-mediated knockout of *RRAGA*. The associated genomic changes in the two *RRAGA* alleles and the resulting changes in the RagA protein are shown. **(b)** CRISPR/Cas9-mediated knockout of *RRAGB*. The associated genomic changes in *RRAGB* are shown. **(c)** CRISPR/Cas9-mediated knockout of *RRAGC*. The associated genomic changes in *RRAGC* and the resulting changes in the RagC protein are shown. **(d)** CRISPR/Cas9-mediated knockout of *RRAGD*. The associated genomic changes in *RRAGD* are shown. Source data

**Extended Data Fig. 2:**
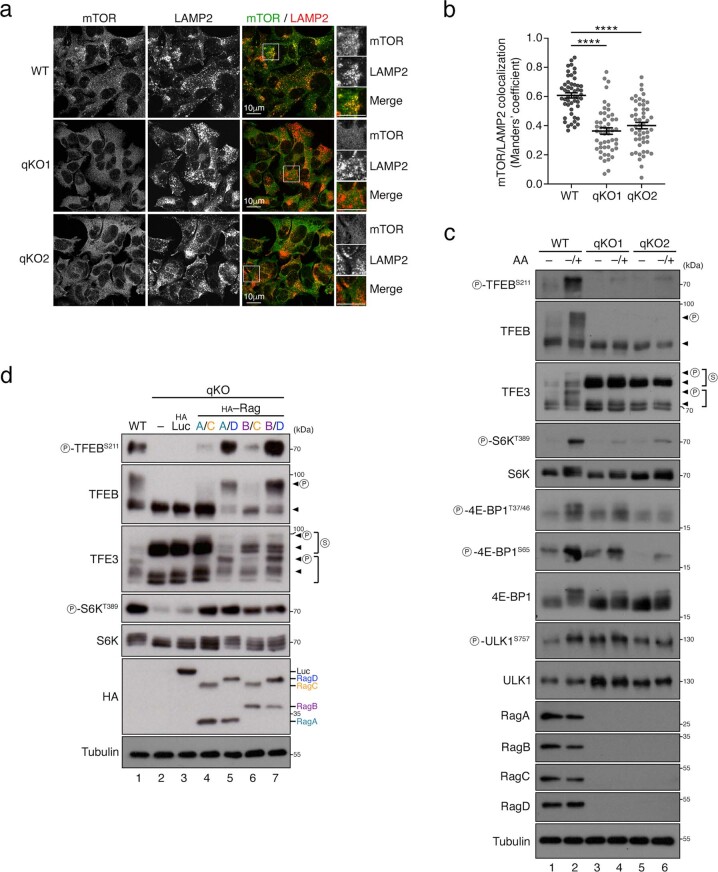
Characterization of the HEK293FT qKO and Rag dimer reconstituted cell lines. **(a-b)** Two independent quadruple RagA-D knockout (qKO) HEK293FT clones show no lysosomal accumulations of mTOR. Colocalization analysis of mTOR with LAMP2 (lysosomal marker) in WT and qKO cells (clones qKO1, qKO2), using confocal microscopy. Magnified insets shown to the right. Scale bars = 10 μm (a). Quantification of mTOR/LAMP2 colocalization from n = 50 individual cells per condition from a representative experiment out of 2 independent replicates is shown in (b). Data shown as mean ± SEM. **** p<0.001. **(c)** Genetic ablation of all four Rags blunts mTORC1 reactivation by amino acids. Immunoblots with lysates from HEK293FT WT and qKO cells (clones qKO1, qKO2), treated with media containing or lacking AA, in starvation (–) or add-back (–/+) conditions, probed with the indicated antibodies. Arrowheads indicate bands corresponding to different protein forms, when multiple bands are present. P: phosphorylated form; S: SUMOylated form. n = 2 independent experiments. **(d)** Reconstitution of qKO cells with different Rag dimers reveals qualitative differences in the regulation of mTORC1. Immunoblots with the indicated antibodies using lysates from HEK293FT WT and qKO cells stably expressing comparable amounts of the four different Rag dimer combinations, or Luciferase (Luc) as a negative control, as HA-tagged proteins. Arrowheads indicate bands corresponding to different protein forms, when multiple bands are present. P: phosphorylated form; S: SUMOylated form. n = 3 independent experiments. Source numerical data and unprocessed blots are available in Source Data. Source data

Although mammalian cells express four Rag proteins (RagA–D) from four distinct genes (*RRAGA*, *RRAGB*, *RRAGC* and *RRAGD*), non-mammal vertebrates (for example, frogs and fishes) lack a second ‘small’ Rag gene, while lower organisms (for example, flies, worms and yeast) have only one ‘small’ and one ‘large’ Rag gene, primarily corresponding to the mammalian RagA and RagC (Fig. 1a). The duplication and sequence diversification of Rag genes in mammals suggest that RagB and RagD may have acquired distinct functions, compared with the ancestral RagA and RagC. Because the Rags function as obligate heterodimers, four possible Rag combinations exist. To investigate whether different Rag dimers have equivalent or diverse functions in the regulation of mTORC1 and AA signalling, we first generated a RagA–D quadruple knock-out (qKO) HEK293FT cell line, using clustered regularly interspaced short palindromic repeats (CRISPR)/Cas9 gene-editing methods (Extended Data Fig. 1a–d). Consistent with the well-known role of the Rags in recruiting mTOR to the lysosomal surface when AAs are abundant^2,9,12,29^, two independent Rag qKO clones demonstrated diminished lysosomal mTOR accumulations (Extended Data Fig. 2a,b) and blunted mTORC1 re-activation upon AA re-supplementation, as assessed by phosphorylation of TFEB, TFE3, S6K, 4E-BP1 and ULK1, five direct mTORC1 substrates (Extended Data Fig. 2c).

**Extended Data Fig. 3:**
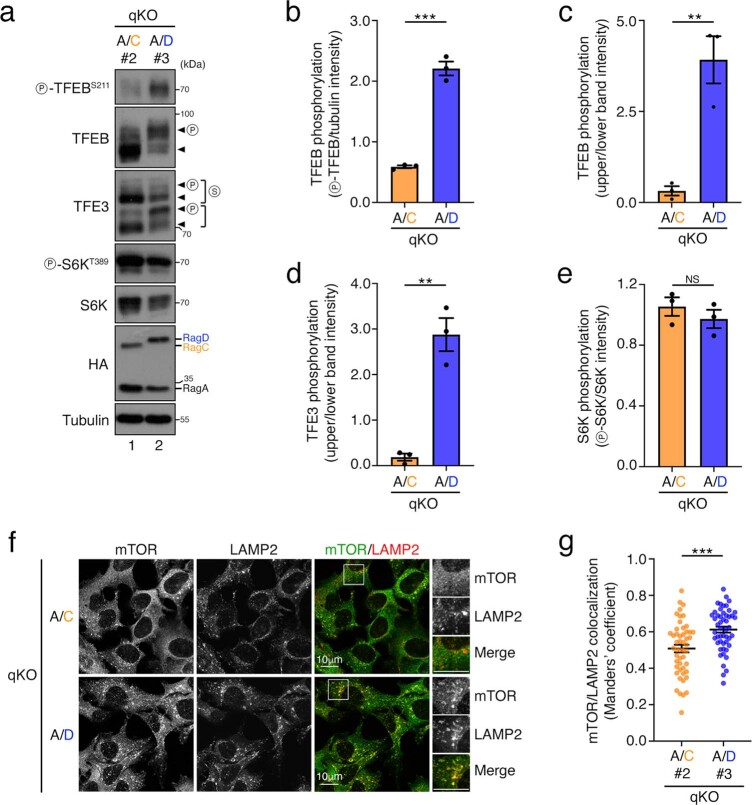
Analysis of independent RagA/C- and RagA/D-expressing clones. **(a-e)** Immunoblots with lysates from qKO HEK293FT cells stably expressing HA-tagged RagA/C or RagA/D, grown under basal, AA-replete culture conditions, probed with the indicated antibodies. Arrowheads indicate bands corresponding to different protein forms, when multiple bands are present. P: phosphorylated form; S: SUMOylated form (a). Quantification of TFEB, TFE3 and S6K phosphorylation from (a), shown in (b-c), (d) and (e), respectively. Data in graphs shown as mean ± SEM. ** p<0.01, *** p<0.005. n = 3 independent experiments. **(f-g)** Colocalization analysis of mTOR with LAMP2 (lysosomal marker) in qKO HEK293FT cells stably expressing RagA/C or RagA/D, grown under basal, AA-replete culture conditions, using confocal microscopy. Magnified insets shown to the right. Scale bars = 10 μm (f). Quantification of mTOR/LAMP2 colocalization from n = 50 individual cells per condition from a representative experiment out of 3 independent replicates is shown in (g). Source numerical data and unprocessed blots are available in Source Data. Source data

We then reconstituted Rag expression in the qKO cells by stably re-expressing one Rag dimer at a time, thus generating the RagA/C, RagA/D, RagB/C and RagB/D cell lines, or a luciferase (Luc)-expressing line as a negative control. To assess the qualitative differences between the four Rag dimers, we selected monoclonal lines that show comparable Rag dimer expression (see also ‘Stable cell line generation’ in Methods), and tested the phosphorylation status of various mTORC1 substrates (Extended Data Fig. 2d). Strikingly, expression of the different Rag dimers differently affected mTORC1 activity towards its substrates in standard growth conditions: although all four dimers were able to restore S6K phosphorylation (albeit with RagA-containing dimers being slightly more potent than RagB-containing dimers), RagD-containing dimers (RagA/D and RagB/D) showed dramatically stronger phosphorylation of TFEB and TFE3 than RagC-containing dimers (Extended Data Fig. 2d). As the Rags are involved in AA signalling to mTORC1, we then tested the responsiveness of the RagA/C- and RagA/D-expressing cells to AA starvation and re-supplementation, using two independent clones each, and observed substantial differences (Fig. 1b–f and Extended Data Fig. 3a–e): RagA/D-expressing cells showed mTORC1 activity towards all substrates, and responded to AA starvation and re-supplementation similarly to wild-type (WT) cells; in contrast, phosphorylation of TFEB/TFE3 was barely detectable in RagA/C-expressing cells, whereas S6K phosphorylation was comparable to that observed in WT and RagA/D cells grown under nutrient-replete conditions.

Considering that TFEB/TFE3 are phosphorylated by active mTORC1 on the lysosomal surface, we reasoned that the observed signalling differences between RagA/C and RagA/D may be due to differential recruitment of mTORC1 to lysosomes. Indeed, we observed significantly stronger lysosomal accumulation of mTOR in RagA/D-expressing cells, whereas RagA/C-reconstituted cells were considerably less capable of rescuing mTOR localization (Fig. 1g,h and Extended Data Fig. 3f,g). Together, these data suggest that the different Rag dimers demonstrate distinct qualities in the regulation of mTORC1 activity and localization, with RagD-containing dimers favouring its lysosomal recruitment and the phosphorylation of its lysosomal substrates.

### RagC and RagD differentially regulate lysosomal biogenesis

**Fig. 2:**
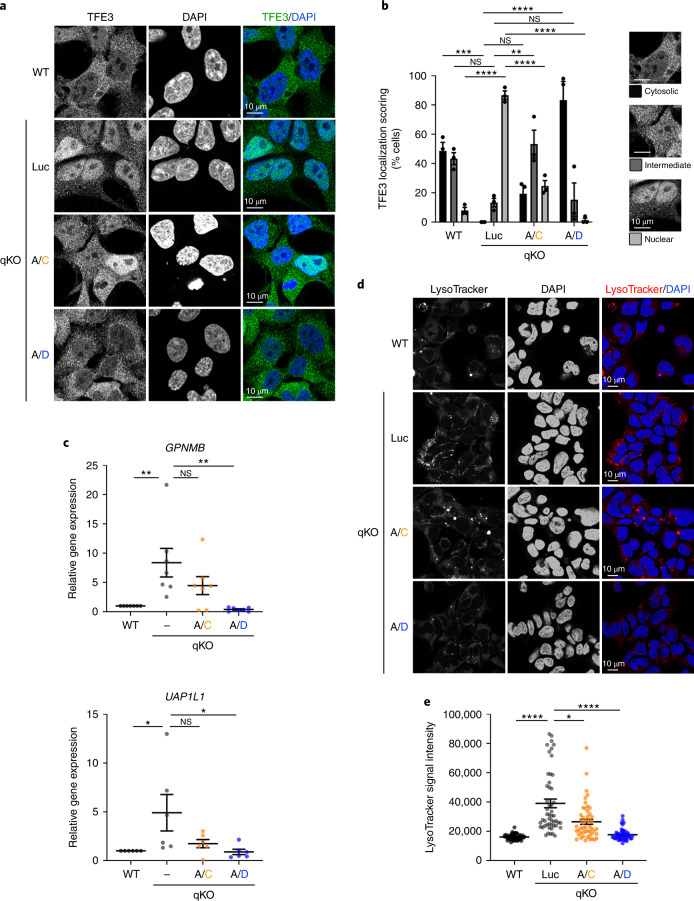
RagC and RagD differentially regulate TFE3 localization, target gene expression and lysosomal biogenesis. **a**, TFE3 localization analysis in HEK293FT WT or qKO cells, stably expressing HA-tagged RagA/C, RagA/D or Luc as a negative control, using confocal microscopy. Nuclei stained with DAPI. Magnified insets shown to the right. Scale bars, 10 μm. *n* = 3 independent experiments. **b**, Scoring of TFE3 localization from **a**. Individual cells were scored for nuclear, intermediate or cytoplasmic TFE3 localization, as indicated in the example images. Scale bars, 10 μm. *n* = 3 independent experiments. **c**, Expression analysis of the TFE3 target genes *GPNMB* and *UAP1L1* in HEK293FT WT, qKO cells or qKOs stably expressing HA-tagged RagA/C or RagA/D. *n*_*GPNMB*_ = 7 independent experiments; *n*_*UAP1L1*_ = 6 independent experiments. **d**, LysoTracker staining in HEK293FT WT, qKO cells or qKOs stably expressing HA-tagged RagA/C or RagA/D. **e**, Quantification of LysoTracker signal intensity from *n* = 50 individual cells per condition from a representative experiment out of three independent replicates. Data in graphs shown as mean ± s.e.m. *P < 0.05, **P < 0.01, ***P < 0.005, ****P < 0.001. Source numerical data are available in source data. Source data

The TFEB/TFE3 transcription factors regulate lysosome biogenesis and autophagy via controlling gene expression in response to nutrient starvation and mTORC1 inhibition^30–32^. Their subcellular localization is controlled by mTORC1: under nutrient-replete conditions, TFEB/TFE3 are recruited to the lysosomal surface in a Rag-dependent manner^33^, where they get phosphorylated by mTORC1, a modification that causes their cytoplasmic sequestration. In contrast, when mTORC1 is inactivated, dephosphorylation of TFEB/TFE3 leads to their re-localization to the nucleus where they promote target gene expression^34–36^. We therefore sought to investigate how TFEB/TFE3 function is influenced by expression of the RagA/C and RagA/D dimers. As expected, endogenous TFE3 showed predominantly nuclear localization in qKO cells, whereas it was mostly cytoplasmic in control cells (Fig. 2a). Consistent with the effects of the two Rag dimers in its phosphorylation, RagA/D expression in qKO cells was able to fully reverse TFE3 localization, whereas RagA/C only partially rescued the nuclear localization phenotype (Fig. 2a,b). The changes in TFE3 localization were further accompanied by changes in the expression of TFEB/TFE3 target genes, such as transmembrane glycoprotein NMB (*GPNMB*) and UDP-*N*-acetylhexosamine pyrophosphorylase-like protein 1 (*UAP1L1*) (refs. ^30,37–42^) (Fig. 2c), and in lysosomal biogenesis, as indicated by LysoTracker staining (Fig. 2d,e): while the increases in gene expression and lysosomal signal observed in qKO cells were completely rescued in RagA/D-expressing cells, RagA/C was much less potent (Fig. 2c–e). In sum, the signalling differences between RagC and RagD in the regulation of mTORC1 translate into functional differences in gene expression and lysosome biogenesis in cells.

### Enhanced association of RagD with lysosomes via p18/LAMTOR1

**Fig. 3:**
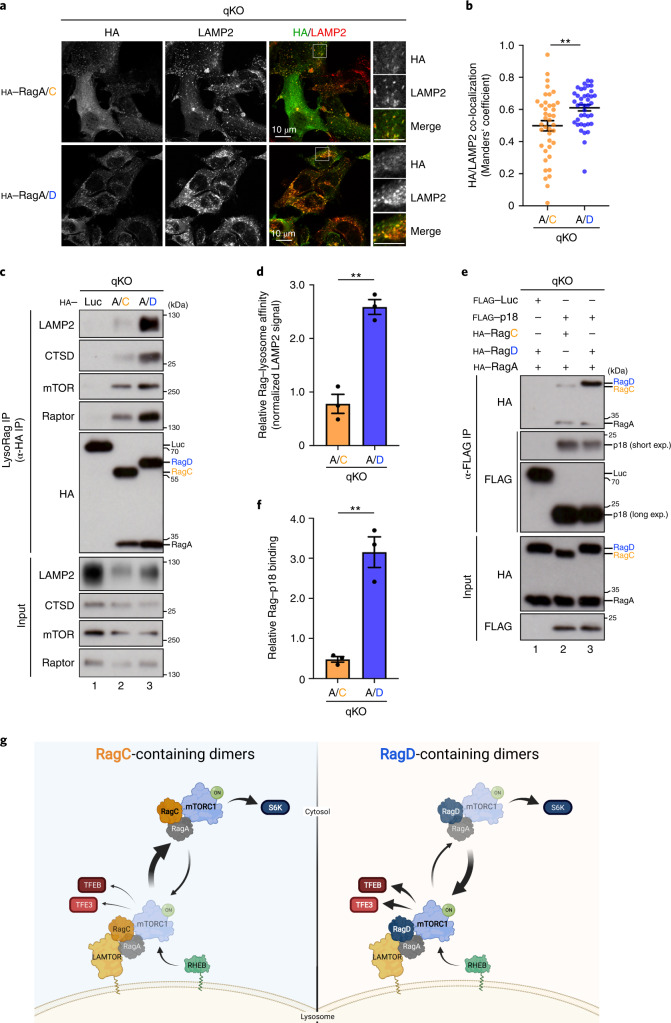
RagD shows higher affinity to p18 and associates with lysosomes more strongly than RagC. **a**, Co-localization analysis of stably expressed HA-tagged RagA/C or RagA/D dimers with LAMP2 (lysosomal marker) in HEK293FT qKO cells, using confocal microscopy. Magnified insets shown to the right. Scale bars, 10 μm. **b**, Quantification of HA/LAMP2 co-localization from *n* = 40 individual cells per condition from a representative experiment out of three independent replicates. **c**, LysoRag IP experiments in HEK293FT qKO cells stably expressing HA-tagged RagA/C, RagA/D or Luc as a negative control. Intact lysosomes were immunopurified by HA–Rag IPs under native conditions and the presence of LAMP2, CTSD, mTOR and Raptor proteins in the lysosomal fractions was analysed by immunoblotting. **d**, Quantification of relative Rag–lysosome affinity. *n* = 3 independent experiments. **e**, p18/LAMTOR1 binds more strongly to RagD, compared with RagC. Co-IP experiments in HEK293FT qKO cells, transiently expressing FLAG-tagged p18 or Luc as negative control, and HA-tagged RagA with RagC or RagD. Binding of the Rags to p18 was analysed by immunoblotting. **f**, Quantification of relative Rag-p18 binding. *n* = 3 independent experiments. **g**, Working model for the differential regulation of mTORC1 by RagC- or RagD-containing dimers. RagD-containing dimers show stronger binding to p18/LAMTOR1, lysosomal localization, lysosomal recruitment of mTORC1, and phosphorylation of the TFE3/TFEB mTORC1 substrates. In contrast, RagC-containing dimers bind much less to p18, localize less to lysosomes and are less potent in recruiting mTORC1 to lysosomes to phosphorylate TFE3/TFEB. Both complexes are similarly capable of driving S6K phosphorylation. See main text for details. Created with BioRender.com. Data in graphs shown as mean ± s.e.m. **P < 0.01. Source numerical data and unprocessed blots are available in source data. Source data

**Extended Data Fig. 4:**
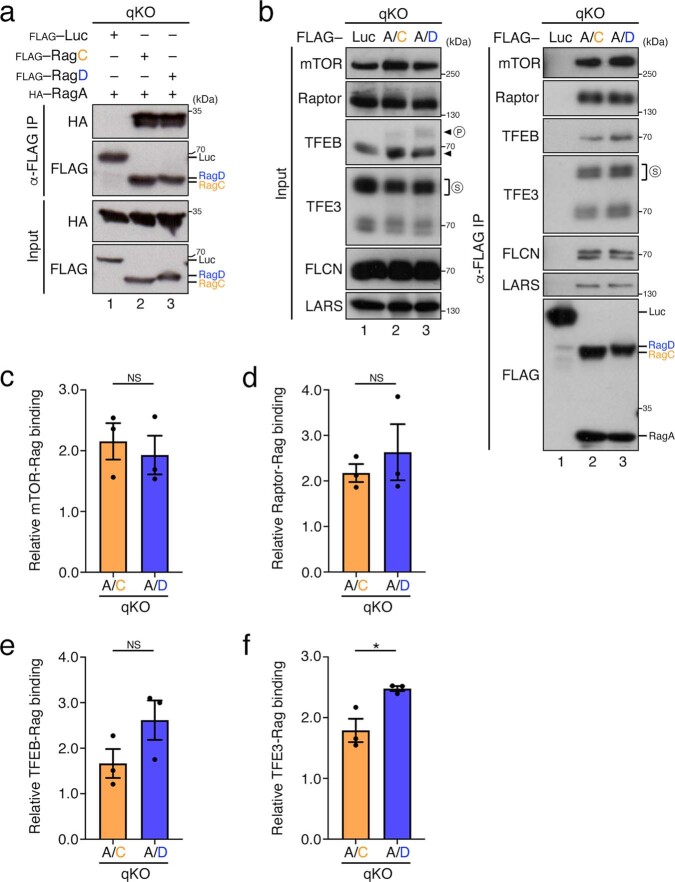
Interaction properties of RagC- and RagD-containing dimers. **(a)** RagC and RagD bind similarly to RagA. Co-immunoprecipitation experiments in HEK293FT qKO cells, transiently expressing HA-tagged RagA with FLAG-tagged RagC, RagD, or Luciferase (Luc) as negative control. Binding of RagA to RagC or RagD was analyzed by immunoblotting as indicated. n = 2 independent experiments. **(b-f)** Co-immunoprecipitation experiments in HEK293FT qKO cells transiently expressing FLAG-tagged RagA/C, RagA/D, or Luciferase (Luc) as a negative control. Binding of the Rags to the indicated proteins was analyzed by immunoblotting. P: phosphorylated protein form; S: SUMOylated protein form(s) (b). Quantification of Rag binding to mTOR, Raptor, TFEB, and TFE3, shown in (c), (d), (e), (f), respectively. n = 3 independent experiments. Data in graphs shown as mean ± SEM. * p<0.05. Source numerical data and unprocessed blots are available in Source Data. Source data

We then aimed to investigate the underlying cause for the functional differences between RagC- and RagD-containing dimers. The presence of RagC or RagD did not influence the stability of the Rag heterodimer as both ‘large’ Rags were equally capable of binding to RagA (Extended Data Fig. 4a), consistent with previous reports^43^. Next, driven by the observation that RagD is more potent than RagC in recruiting mTOR to lysosomes, we reasoned that the localization of these Rags themselves may also differ. Indeed, in co-localization/confocal microscopy experiments, RagD-containing dimers showed significantly stronger lysosomal localization than RagC-containing dimers (Fig. 3a,b). To independently confirm these findings, we developed a biochemical approach, which we named LysoRag IP, that is a modified version of the Lyso-IP method previously established by others^44^. Using the qKO cell lines that stably express haemagglutinin (HA)-tagged RagA/C or RagA/D (or an unrelated protein as control), we performed detergent-free cell lysis and co-immunoprecipitated intact lysosomes under native conditions with HA-tagged Rags as bait. With this method, the amount of lysosomes that is pulled down with the Rags is indicative of the relative affinity of each Rag dimer to the lysosomes. In agreement with our microscopy studies, RagD-containing dimers specifically co-purified more lysosomes, compared with RagC-containing dimers, as indicated by the lysosome-associated membrane glycoprotein 2 (LAMP2) and cathepsin D (CTSD) lysosomal markers (Fig. 3c,d). Accordingly, the lysosomal fraction from RagA/D samples also contained higher levels of mTORC1 components, that is, mTOR and Raptor, albeit the differences to the RagA/C samples were not as dramatic as those for lysosomal markers (Fig. 3c).

As the Rags are only indirectly tethered to the lysosomal surface, via binding to the LAMTOR complex, we then tested if the Rag–LAMTOR interaction is the underlying cause for the increased lysosomal tethering of RagD. Indeed, co-immunoprecipitation (co-IP) experiments with exogenously expressed FLAG-tagged p18/LAMTOR1 (or an unrelated FLAG-tagged protein as control) and HA-tagged RagA/C or RagA/D in qKO cells, showed that RagD bound much more strongly to p18 (Fig. 3e,f), consistent with a previous report^28^. Interestingly, their interaction with mTORC1 components (mTOR, Raptor), the lysosomal mTORC1 substrates (TFEB, TFE3) or the upstream RagC/D regulators (FLCN, LARS) was comparable between RagC- and RagD-containing dimers (Extended Data Fig. 4b–f), suggesting that the increased RagD-p18 binding is the primary cause for the enhanced mTORC1 recruitment to lysosomes and the phosphorylation of TFEB/TFE3 (Fig. 3g, right). In contrast, RagC-containing dimers localize less strongly to lysosomes, owing to weaker binding to p18, but are still capable of binding mTORC1 and inducing the phosphorylation of cytoplasmic substrates, such as S6K (Fig. 3g, left). These data favour a model where RagC and RagD are qualitatively different from each other and define substrate specificity downstream of mTORC1, thereby differentially regulating mTORC1 functions such as lysosome biogenesis.

### The RagC/D terminal regions define their distinct properties

**Fig. 4:**
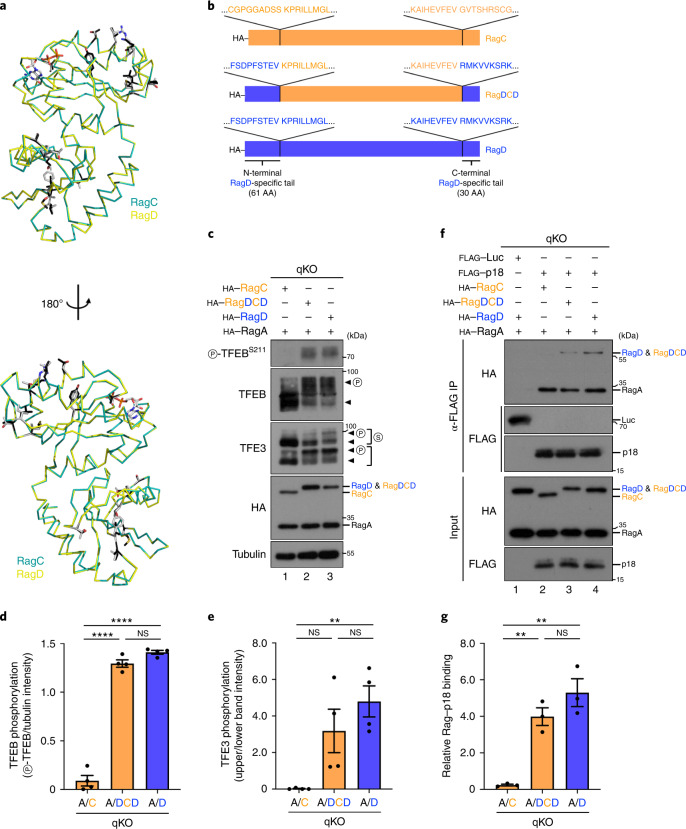
Differences in the N- and C-terminal RagD regions are responsible for its differential behaviour, compared with RagC. **a**, Superposition of the structure of RagC (from PDBID: 6S6D; shown in cyan) with RagD (modelled; shown in yellow) shows high structural similarity between the two structures. Side chains of variable positions shown as dark grey (RagC) or light grey (RagD) sticks. **b**, Schematic representation of HA-tagged RagC, RagD and the RagDCD chimaera, in which the N- and C-terminal tails of RagC were replaced with those of RagD. The AA sequences around the fusion points are shown as insets. **c**, Immunoblots with lysates from HEK293FT qKO cells stably expressing HA-tagged RagA with RagC, RagD or the RagDCD chimaera, probed with the indicated antibodies. Arrowheads indicate bands corresponding to different protein forms, when multiple bands are present. P, phosphorylated form; S, SUMOylated form. **d**,**e**, Quantification of TFEB (**d**) and TFE3 (**e**) phosphorylation. *n* = 4 independent experiments. **f**, Co-IP experiments in HEK293FT qKO cells transiently expressing FLAG-tagged p18 or Luc as control, and HA-tagged RagA with RagC, RagD or the RagDCD chimaera. Binding of p18 to the Rags was analysed by immunoblotting. **g**, Quantification of relative Rag-p18 binding. *n* = 3 independent experiments. Data in graphs shown as mean ± s.e.m. **P < 0.01, ****P < 0.001. Source numerical data and unprocessed blots are available in source data. Source data

**Extended Data Fig. 5:**
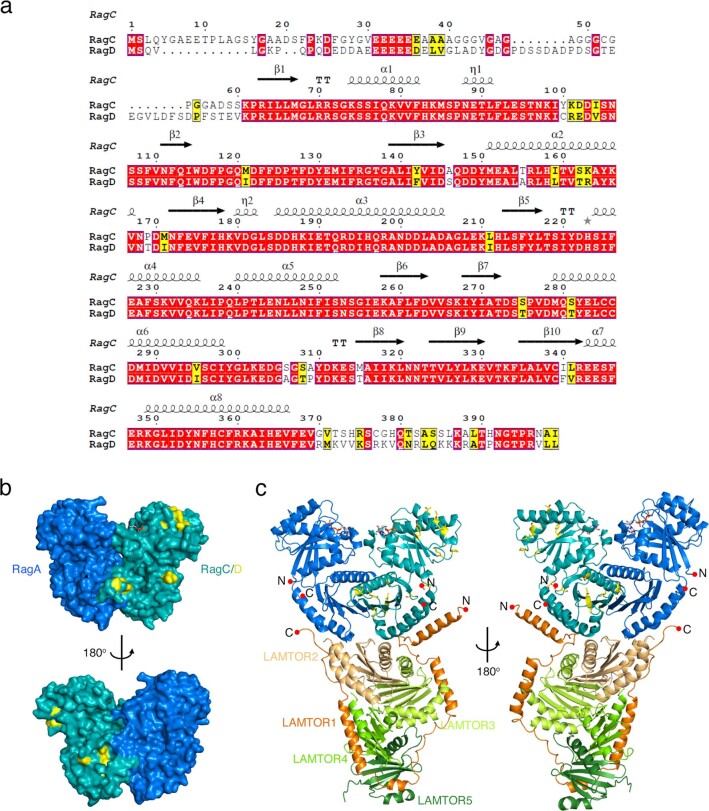
Structural comparison between RagC and RagD suggests main differences localize to the unstructured N- and C-terminal tails. **(a)** Structure-based sequence alignment of RagC and RagD prepared with ESPript. Similar and identical residues are marked by yellow and red boxes, respectively. Secondary structure assignment is based on PDBID: 6S6D. **(b)** Minimal surface residue differences between RagC and RagD. Surface representation of the RagA (blue) / RagC (cyan) heterodimer (PDBID: 6S6D). Variable positions between RagC and RagD are coloured yellow. **(c)** No residue differences between the RagC and RagD structures localize at the Rag dimer / LAMTOR complex interface. Model of the RagA (blue) / RagC (cyan) heterodimer in the active conformation (PDBID: 6S6D) bound to the pentameric LAMTOR1-5 complex (PDBID: 6EHP). Variable positions between RagC and RagD are shown as yellow sticks. The ultimate residues that could be modelled at the N- and C-termini of the RagA, RagC and LAMTOR1 in the published structures are also labelled (N, C). Source data

The RagC and RagD paralogues share ~80% AA sequence identity^21^, with the majority of differences between the two proteins localizing to the unstructured N- and C-terminal regions (Extended Data Fig. 5a). To investigate which are the responsible parts for the functional differences between RagC and RagD, we first modelled RagD by introducing substitutions in the RagC structure resolved previously (PDBID: 6S6D)^45^. Of note, this ‘core’ structure does not include the disordered, variable N- and C-terminal tails of RagC (residues 1–58 and 370–399, respectively). As expected from their AA sequence alignment (Extended Data Fig. 5a), a surface representation of the variable positions between the RagC and RagD cores (as heterodimers with RagA) showed minimal surface residue differences (Extended Data Fig. 5b), none of which localizes at the Rag dimer interface with the LAMTOR complex (PDBID: 6EHP)^46^ (Extended Data Fig. 5c). Accordingly, superposition of the core structure of RagC with the respective RagD model showed very high similarity between the two structures (Fig. 4a).

To experimentally test if the N- and C-terminal unstructured tails of RagC and RagD are the cause of their functional differences, we then generated qKO cells stably expressing a ‘RagDCD’ chimaeric protein, in which the N-terminal 60 AA and the C-terminal 30 AA of RagC were replaced by the respective RagD tails (Fig. 4b). Notably, despite containing the complete core region of RagC, the RagDCD chimaera closely resembled the properties of RagD, showing elevated TFEB/TFE3 phosphorylation (Fig. 4c–e) and enhanced binding to exogenously expressed FLAG-tagged p18 in co-IP experiments (Fig. 4f,g). These structural and biochemical analyses suggest that the differences in the N- and C-terminal RagD regions are responsible for its differential behaviour, compared with RagC.

### Cancer-associated RagC mutants upregulate lysosomal mTORC1

**Fig. 5:**
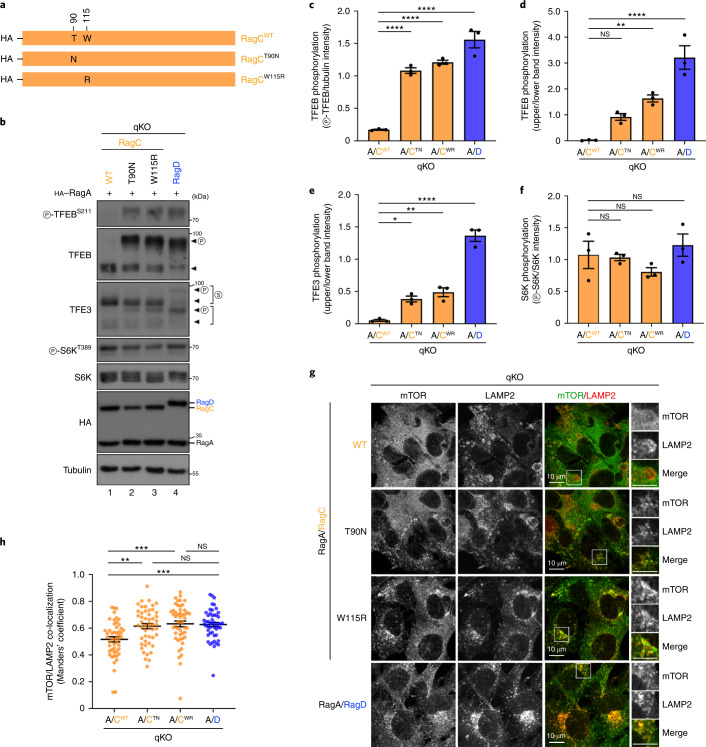
Cancer-associated RagC mutations enhance TFE3/TFEB phosphorylation and mTOR lysosomal recruitment. **a**, Schematic representation of HA-tagged WT RagC and the cancer-related T90N, W115R RagC point mutants. **b**, Immunoblots with lysates from HEK293FT qKO cells stably expressing HA-tagged RagA with WT RagC or RagD, or the T90N, W115R RagC mutants, probed with the indicated antibodies. Arrowheads indicate bands corresponding to different protein forms, when multiple bands are present. P, phosphorylated form; S, SUMOylated form. **c**–**f**, Quantification of TFEB (**c** and **d**), TFE3 (**e**) and S6K (**f**) phosphorylation from **b**. *n* = 3 independent experiments. **g**, Co-localization analysis of mTOR with LAMP2 (lysosomal marker) in HEK293FT qKO cells stably expressing HA-tagged RagA with the RagC proteins shown in **a** or RagD, using confocal microscopy. Magnified insets shown to the right. Scale bars, 10 μm. **h**, Quantification of mTOR/LAMP2 co-localization from *n* = 50 individual cells per condition from a representative experiment out of three independent replicates. Data in graphs shown as mean ± s.e.m. *P < 0.05, **P < 0.01, ***P < 0.005, ****P < 0.001. Source numerical data and unprocessed blots are available in source data. Source data

**Extended Data Fig. 6:**
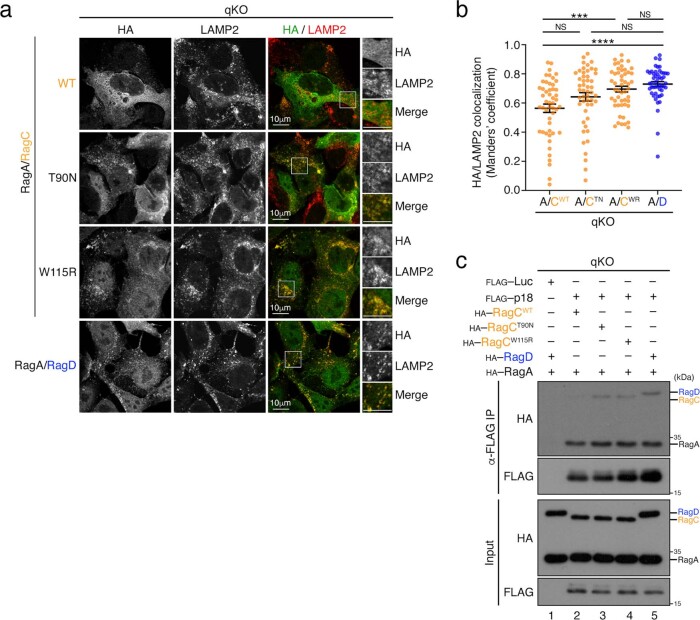
Cancer-associated RagC mutations show increased lysosomal localization and p18 binding. **(a-b)** Colocalization analysis between the Rags and LAMP2 (lysosomal marker) in HEK293FT qKO cells stably expressing HA-tagged RagA with WT RagC, RagD, or the T90N and W115R RagC mutants, using confocal microscopy. Magnified insets shown to the right. Scale bars = 10 μm (a). Quantification of HA/LAMP2 colocalization from n = 50 individual cells per condition from a representative experiment out of 2 independent replicates is shown in (b). Data shown as mean ± SEM. *** p<0.005, **** p<0.001. **(c)** The cancer-associated RagC mutants bind more strongly to p18/LAMTOR1, compared to wild-type RagC. Co-immunoprecipitation experiments in HEK293FT qKO cells, transiently expressing FLAG-tagged p18 or Luciferase (Luc) as negative control, and HA-tagged RagA with WT RagC, RagD, or the T90N and W115R RagC mutants. Binding of the Rags to p18 was analyzed by immunoblotting as indicated. n = 2 independent experiments. Source numerical data and unprocessed blots are available in Source Data. Source data

Genetic analyses have previously identified activating mutations in *RRAGC* in patients with follicular lymphoma^45,47,48^, highlighting the importance of the dysregulation of RagC activity in human disease. Two such mutants, RagC^T90N^ and RagC^W115R^, were previously described to enhance mTORC1 activity when overexpressed in HEK293T cells (assessed by S6K phosphorylation)^47^. Given that WT RagC shows very weak activation of mTORC1 towards TFEB/TFE3, we wondered if the oncogenicity of these mutants also involves aberrant activation of this pathway. Indeed, compared with RagC^WT^-expressing cells, qKO cells stably expressing each activating RagC mutant (Fig. 5a) showed strongly elevated TFEB/TFE3—but not S6K—phosphorylation (Fig. 5b–f), accompanied by enhanced lysosomal recruitment of mTOR (Fig. 5g,h), largely resembling the behaviour of RagD. Accordingly, the RagC mutant proteins localized more strongly to lysosomes (Extended Data Fig. 6a,b), probably due to increased affinity to p18 (Extended Data Fig. 6c).

### The RagA/B paralogues differently control mTORC1 in starvation

**Fig. 6:**
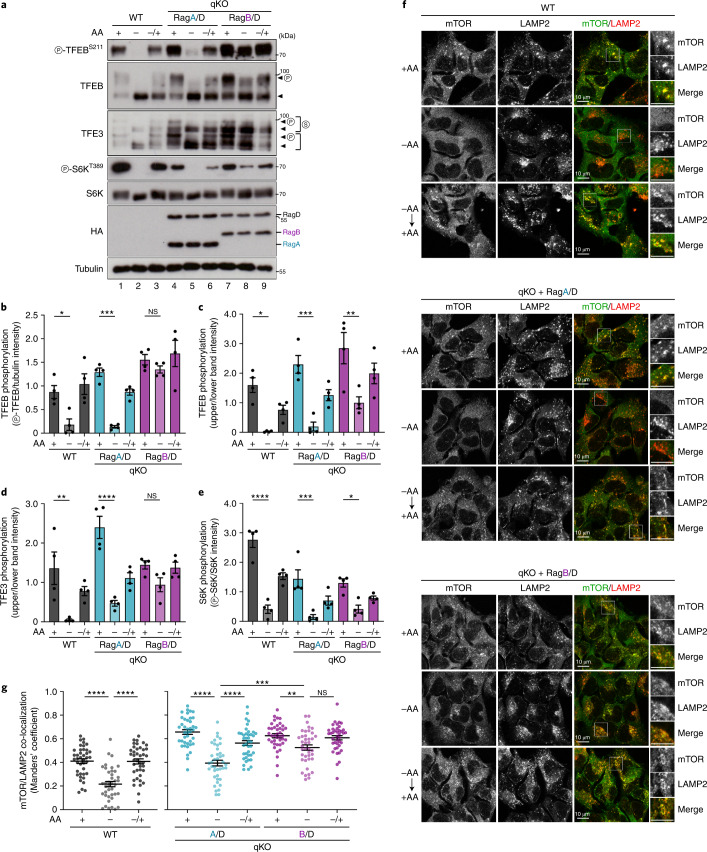
The RagA and RagB paralogues differentially control mTORC1 activity upon AA starvation. **a**, Immunoblots with lysates from HEK293FT WT, or qKO cells stably expressing RagA/D or RagB/D, treated with medium containing (+) or lacking (–) AA, in basal (+), starvation (–) or add-back (–/+) conditions, probed with the indicated antibodies. Arrowheads indicate bands corresponding to different protein forms, when multiple bands are present. P, phosphorylated form; S, SUMOylated form. **b**–**e**, Quantification of TFEB (**b** and **c**), TFE3 (**d**) and S6K (**e**) phosphorylation from the blots shown in **a**. *n* = 4 independent experiments. **f**, Co-localization analysis of mTOR with LAMP2 (lysosomal marker) in HEK293FT WT or qKO cells stably expressing RagA/D or RagB/D, using confocal microscopy. Magnified insets shown to the right. Scale bars, 10 μm. **g**, Quantification of mTOR/LAMP2 co-localization from *n* = 40 individual cells per condition from a representative experiment out of two independent replicates. Data in graphs shown as mean ± s.e.m. *P < 0.05, **P < 0.01, ***P < 0.005, ****P < 0.001. Source numerical data and unprocessed blots are available in source data. Source data

**Extended Data Fig. 7:**
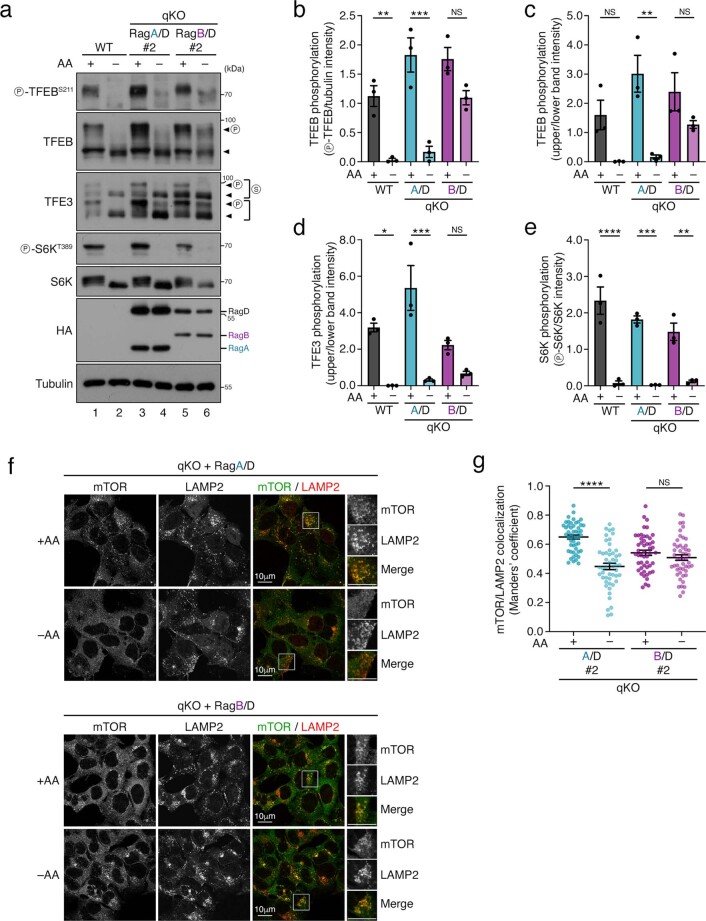
Analysis of independent RagA/D- and RagB/D-expressing clones. **(a-e)** Immunoblots with lysates from HEK293FT WT or qKO cells stably expressing RagA/D or RagB/D, treated with media containing (+) or lacking (–) AA, probed with the indicated antibodies. Arrowheads indicate bands corresponding to different protein forms, when multiple bands are present. P: phosphorylated form; S: SUMOylated form (a). Quantification of TFEB, TFE3 and S6K phosphorylation from the blots in (a), shown in (b-c), (d), and (e), respectively. n = 3 independent experiments. **(f-g)** Colocalization analysis of mTOR with LAMP2 (lysosomal marker) in HEK293FT WT or qKO cells stably expressing RagA/D or RagB/D, using confocal microscopy. Magnified insets shown to the right. Scale bars = 10 μm (f). Quantification of mTOR/LAMP2 colocalization from n = 50 individual cells per condition from a representative experiment out of 3 independent replicates is shown in (g). Data in all graphs shown as mean ± SEM. * p<0.05, ** p<0.01, *** p<0.005, **** p<0.001. Source numerical data and unprocessed blots are available in Source Data. Source data

RagB is a mammal-specific paralogue of RagA (Fig. 1a), with the two Rags showing very high AA sequence identity (90%). Our initial analysis of mTORC1 activity in the Rag-dimer-reconstituted qKO lines grown under basal, nutrient-replete culture conditions did not show robust differences between RagA- and RagB-containing dimers (Extended Data Fig. 2d). To investigate if RagB is functionally divergent from RagA under different nutritional conditions, we compared mTORC1 activity in qKO cells stably expressing RagA or RagB in complex with RagD (instead of RagC, to be able to also assess effects on TFEB/TFE3 phosphorylation), cultured under basal, starvation or AA re-addition conditions. These experiments, analysing two independent clones for each Rag combination, revealed that mTORC1 activity in RagA/D-expressing cells responds to AA withdrawal and re-supplementation similarly to WT cells (Figs. 1b and 6a–e and Extended Data Fig. 7a–e). Intriguingly, RagB/D expression caused attenuated response to starvation (Fig. 6a–e and Extended Data Fig. 7a–e), as assessed by phosphorylation of TFEB/TFE3 (Fig. 6a–d and Extended Data Fig. 7a–d) and—to a much lesser extent—S6K (Fig. 6a,e and Extended Data Fig. 7a,e). The incomplete mTORC1 inactivation in RagB/D-expressing cells was also reflected in the lysosomal localization of mTOR: whereas it readily de-localized away from lysosomes upon starvation in WT and RagA/D-expressing cells, RagB/D-expressing cells maintained substantial amounts of lysosomal mTOR (Fig. 6f,g and Extended Data Fig. 7f,g).

### Structure–function analysis of RagA/B in AA starvation

**Fig. 7:**
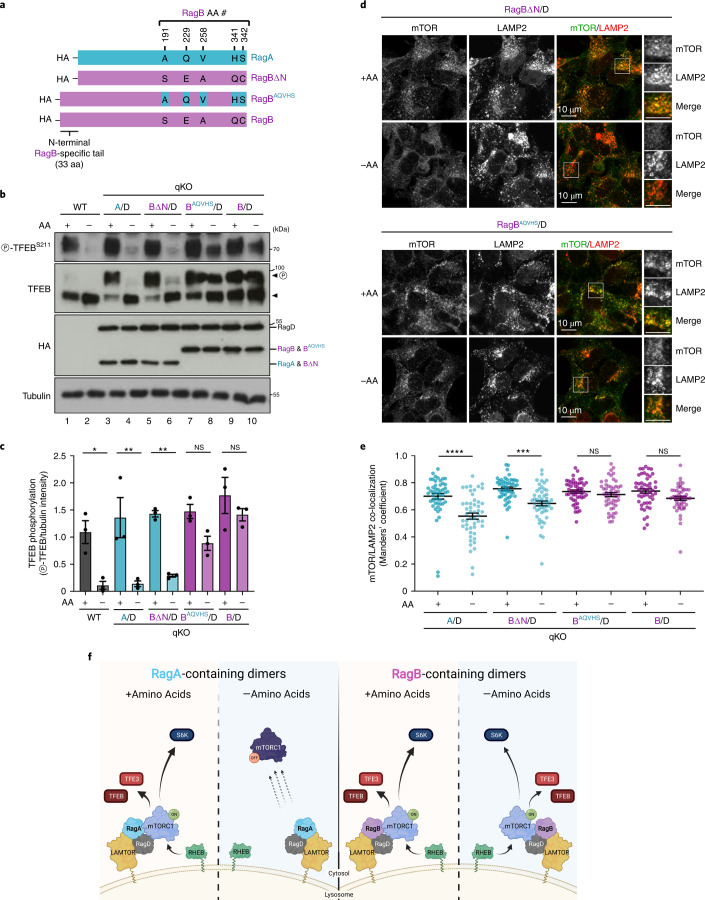
The RagB-specific N-terminal tail is responsible for its differential effect towards mTORC1 upon AA starvation, compared with RagA. **a**, Schematic representation of HA-tagged WT RagA, WT RagB and the RagBΔN, RagB^AQVHS^ chimaeras. **b**, Immunoblots with lysates from HEK293FT qKO cells stably expressing the proteins shown in **a** as dimers with HA-tagged RagD, probed with the indicated antibodies. Arrowheads indicate bands corresponding to different protein forms, when multiple bands are present. P, phosphorylated form. **c**, Quantification of TFEB phosphorylation. *n* = 3 independent experiments. **d**, Co-localization analysis of mTOR with LAMP2 (lysosomal marker) in HEK293FT qKO cells stably expressing HA-tagged RagBΔN or RagB^AQVHS^ as dimers with RagD, treated with medium containing (+) or lacking (–) AA, using confocal microscopy. Magnified insets shown to the right. Scale bars, 10 μm. **e**, Quantification of mTOR/LAMP2 co-localization from *n* = 50 individual cells per condition from a representative experiment out of three independent replicates. **f**, Working model for the differential regulation of mTORC1 by RagA- or RagB-containing dimers, in basal or AA starvation conditions. Whereas RagA-containing dimers allow for mTORC1 de-localization away from lysosomes and for its inactivation upon AA starvation, RagB-containing dimers retain lysosomal and active mTORC1 even in AA starvation conditions. See main text for details. Created with BioRender.com. Data in graphs shown as mean ± s.e.m. *P < 0.05, **P < 0.01, ***P < 0.005, ****P < 0.001. Source numerical data and unprocessed blots are available in source data. Source data

**Extended Data Fig. 8:**
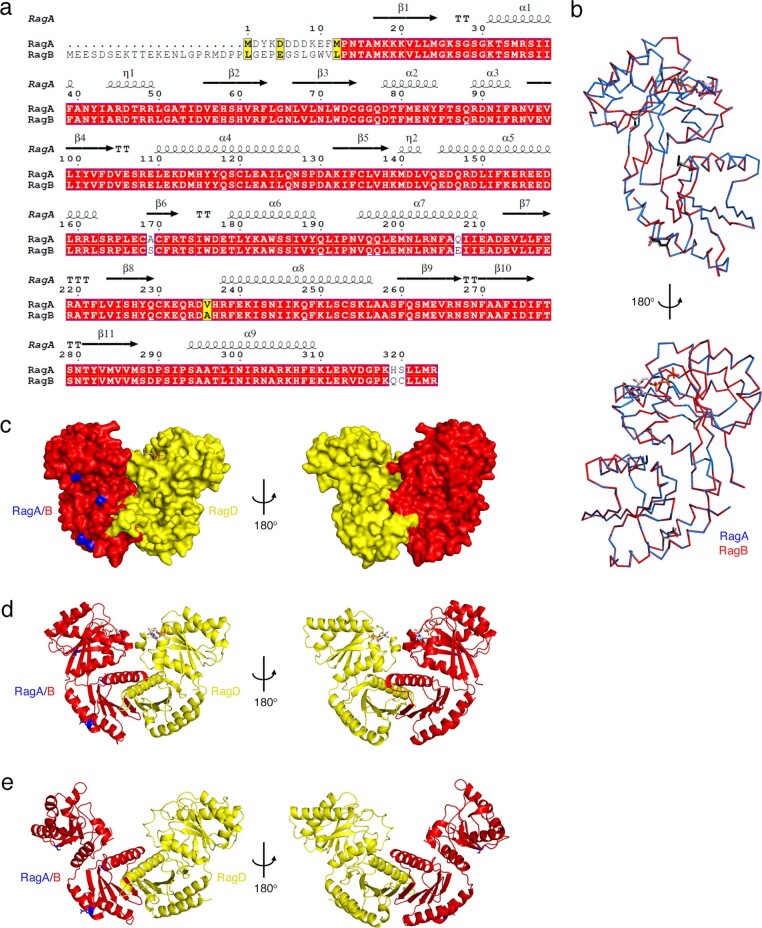
Structural comparison between RagA and RagB suggests main differences localize to the unstructured N-terminal tail. **(a)** Structure-based sequence alignment of RagA and RagB prepared with ESPript. Similar and identical residues are marked by yellow and red boxes, respectively. Secondary structure assignment is based on PDBID: 6S6D. **(b)** Residue differences in the structures of RagA and RagB are not predicted to cause overall structural changes. Superposition of the structure of RagA (from PDBID: 6S6D; shown in blue) with RagB (modelled; shown in red) shows high structural similarity between the two structures. Side chains of variable positions shown as dark grey (RagA) or light grey (RagB) sticks. **(c)** Minimal surface residue differences between RagA and RagB. Surface representation of the model of RagB (red) / RagD (yellow) heterodimer in the active conformation (modelled based on PDBID: 6S6D). Variable positions between RagA and RagB are coloured blue. **(d)** Ribbon representation of the model of the RagB (red) / RagD (yellow) heterodimer in the active conformation (modelled based on PDBID: 6S6D). Variable positions between RagA and RagB are coloured blue and side chains are shown as sticks. **(e)** Same as in (d), but for the inactive RagB/D dimer (modelled based on PDBID: 6ULG).

The AA sequence differences between the RagA/B paralogues map to two regions: the RagB-specific disordered N-terminal tail, spanning 33 AA; and five AA substitutions in the folded RagA/B ‘body’ (Fig. 7a and Extended Data Fig. 8a). To look into the structural differences between RagA and RagB that may explain their functional divergence, we used the active RagA/RagC dimer structure (PDBID: 6S6D)^45^ and introduced residue substitutions to model the respective RagB/RagD dimer. The few RagA/B differences predicted no structural changes between the RagA structure and the RagB model, both as dimers with RagD (Extended Data Fig. 8b–d). The same was true when comparing the two ‘small’ Rags in their inactive conformation (based on PDBID: 6ULG) (Extended Data Fig. 8e). Therefore, the structural comparison between RagA and RagB suggests that functional differences are probably encoded by the variable, unstructured N-terminal tail of RagB.

We then generated qKO cell lines, stably expressing RagB mutants that resemble the RagA structural characteristics, either by removing the N-terminal RagB tail (‘RagBΔN’) or by substituting the five variable residues in the RagB ‘body’ with those of RagA (‘RagB^AQVHS^’), as HA-tagged proteins (Fig. 7a), together with RagD. Both mutants were described previously^2^. Whereas cells expressing RagBΔN responded to AA starvation similarly to WT or RagA/D-expressing cells, RagB^AQVHS^ expression resembled more closely the partial insensitivity to AA removal observed with full-length, RagB^WT^-containing dimers, in terms of both mTORC1 activity (Fig. 7b,c) and lysosomal localization (Fig. 7d,e). These data confirm that the RagB-specific N-terminal tail is responsible for the incomplete response of mTORC1 to AA starvation.

In sum, the findings presented here underscore the functional divergence between the RagA and RagB paralogues, with the former responding fully to AA removal to dynamically regulate mTORC1 localization and activity, and the latter retaining lysosomal and active mTORC1 even in starved cells (Fig. 7f).

## Discussion

In most studies, the RagA/B and the RagC/D paralogues are referred to as functionally redundant, despite lack of experimental evidence that supports this statement. On the contrary, several hints in the literature imply that Rag paralogues may possess gene-specific functions. A recent study identified the mTORC1-mediated phosphorylation of RagC on Ser21 as part of an autoregulatory mechanism that fine-tunes mTORC1 activity towards S6K and 4E-BP1 in response to growth factor and AA signalling^49^. Notably, none of the other Rags was found to be phosphorylated under the same conditions^49^, and this RagC phospho-residue is not conserved in the N-terminus of RagD, suggesting that RagC may not be a biological equivalent to RagD. As mentioned above, the LARS GTPase-activating protein (GAP) was shown in another study to bind and regulate RagD—but not RagC—despite their high sequence homology^27,28^. Similarly, the lysosomally localized GATOR1 complex components nitrogen permease regulator 2-like protein (NPRL2) and NPRL3 preferentially bind to RagD—over RagC—in an AA- and GTP/GDP-loading-dependent manner^50^, whereas the mitochondrial threonyl-tRNA synthetase 2 (TARS2) interacts primarily with RagC—but not RagD—in response to threonine availability^51^. Finally, looking into the mechanistic details of the lysosomal recruitment of TSC upon AA starvation, we have previously described strong binding preference of TSC2 to RagA, compared with all other Rags^2^. These experimental observations are in accordance with the classical evolutionary theory of gene duplication and diversification, based on which paralogous genes often participate in distinct regulatory networks and acquire specialized functions^14,15^.

We report here that a Rag dimer code defines the substrate specificity downstream of mTORC1, and its responsiveness to starvation. In particular, RagD appears to be responsible for the regulation of mTORC1 on lysosomes, where it phosphorylates the TFEB/TFE3 transcription factors to control lysosomal biogenesis and autophagy, whereas RagC seems to be more loosely connected to the lysosomal LAMTOR tethering complex and presumably more relevant for the phosphorylation of non-lysosomal mTORC1 substrates like S6K. Although the Rags are traditionally viewed as lysosomal proteins, our data suggest that their relative affinity to lysosomes is variable. Interestingly, early work that identified the LAMTOR–Rag interaction showed that the binding between LAMTOR subunits and a RagA/C dimer is weakened upon AA re-supplementation^23^, suggesting that the lysosomal localization of RagC-containing dimers may dynamically respond to AA availability. Indeed, a more recent study revealed that RagC-containing dimers cycle between the lysosomal surface and the cytosol, with nutrients enhancing its re-localization by weakening the association of the Rags with the lysosomally bound LAMTOR complex^43^. Of note, the cancer-related RagC mutants—that we show here increase TFEB phosphorylation and lysosomal localization of mTOR—were also found to stabilize the RagC–LAMTOR association and reduce cycling of a RagB/C dimer^43^. In sum, we here identify RagC/RagD as substrate- and location-specific regulators of mTORC1. The search for ways to selectively modify their activities will probably provide tools to perturb specific pathways downstream of mTORC1, and dissect the relative contribution of these pathways in conditions where mTOR is dysregulated.

According to the publicly available protein and mRNA expression data (summarized in the Human Protein Atlas webpage; www.proteinatlas.org), no tissues that express exclusively RagA or RagB exist, with RagA generally being expressed at higher levels in most tissues. Therefore, RagA- and RagB-containing dimers probably co-exist in cells, where they may differentially regulate how subpopulations of mTORC1 respond to starvation. While the more abundant RagA-containing dimers would ensure a proper inactivation of mTORC1 when AA levels drop, RagB-containing dimers may be responsible for maintaining a baseline mTORC1 activity tone, to support essential physiological processes even upon starvation. Consistent with such a model, AA-starved cells do not completely shut off protein synthesis or gene expression. For instance, mitochondrial protein synthesis has been reported to actually increase upon starvation^52^. Moreover, the ATF4 transcription factor lies downstream of both mTORC1 and GCN2 signalling and upregulates the expression of specific genes as part of the cellular stress/starvation response^53,54^. Finally, autophagosome and lysosome biogenesis, two processes that require massive upregulation of the constituent proteins—many of which are produced in an mTORC1-activity-dependent manner—are also known to be induced in starved cells. Whether mTORC1 complexes that stay active by binding to RagB-containing dimers on lysosomes contribute to such starvation-induced cellular processes will be important to investigate in the future.

At the molecular level, we show that functional differences between RagA versus RagB and RagC versus RagD are encoded by their variable termini. These regions are unstructured and unlikely to directly influence their GTPase cycle. However, they may provide sites for post-translational modifications or interaction motifs for regulatory proteins, as we report here for p18 preferentially binding to RagD. Accordingly, in an accompanying paper, Figlia et al. reveal that RagB isoforms maintain active mTORC1 in starved neurons or various tumours by inhibiting GATOR1, the RagA/B GTPase activating protein complex^55^.

In addition to mediating the binding of mTORC1 to the lysosomal surface, the Rag GTPases are also necessary for the recruitment of the TFEB/TFE3 transcription factors to lysosomes, where they are phosphorylated by mTORC1. Consequently, Rag- or LAMTOR-mutant cells have non-phosphorylated and constitutively nuclear TFEB/TFE3 (refs. ^32,33,56^). Therefore, the differential behaviour of RagC and RagD towards the regulation of TFEB/TFE3 phosphorylation and localization could be explained by differences in their ability to either recruit and activate the kinase (that is, mTORC1) or function as a lysosomal tether for the substrates (that is, TFEB/TFE3). Our data show that overexpressed RagC- and RagD-containing dimers have similar affinities for TFEB, TFE3, mTOR and Raptor (Extended Data Fig. 4b–f), with the primary difference being the strongly enhanced binding of RagD to p18/LAMTOR1 (Fig. 3e,f), which is the likely cause for their functional divergence.

The TFEB/TFE3 transcription factors are master regulators of a starvation-induced transcriptional programme that controls lysosome biogenesis and autophagy. The importance of this process is underscored by the existence of autoregulatory feedback mechanisms that ensure proper fine-tuning of the Rag–mTORC1–TFEB/TFE3 signalling hub. For instance, TFEB expression was previously shown to be induced by starvation via a positive feedback loop that involves direct TFEB binding to its own promoter^57^. Moreover, TFEB and TFE3 control lysosomal recruitment and activity of mTORC1 by robustly upregulating RagD expression, whereas RagC expression is much less affected^58,59^. Our data expand this model further, showing that the TFEB/TFE3 target, RagD, is—in turn—the key regulator of their phosphorylation, subcellular localization and activity, thus establishing a negative feedback loop to facilitate rapid and robust re-phosphorylation and inactivation of TFEB/TFE3 when AAs are available again, following starvation.

In sum, our work identifies the mammalian Rag GTPases as a unique example of functionally divergent paralogues in the core AA sensing/mTOR signalling pathway. Through evolution, duplication of the ancestral *RRAGA* and *RRAGC* genes and functional diversification of the additional copies has led to four mammalian Rags that form distinct Rag dimers with specialized functions in the regulation of mTORC1 by AAs. Hence, our findings support the existence of a Rag dimer code that adds to the complexity of metabolic signalling in mammalian cells.

## Methods

### Cell culture and treatments

All cell lines were grown at 37 °C, 5% CO_2_. Human female embryonic kidney HEK293FT cells (#R70007, Invitrogen; RRID: CVCL_6911) and the resulting genetically modified cell lines were cultured in high-glucose DMEM (#41965039, Thermo Fisher Scientific), containing 10% foetal bovine serum (FBS) and 1% penicillin–streptomycin. The parental HEK293FT cells were purchased from Invitrogen before the initiation of the project. Their identity was validated by the Multiplex human Cell Line Authentication test (Multiplexion GmbH), which uses a single-nucleotide polymorphism typing approach, and was performed as described at www.multiplexion.de. All cell lines were regularly tested for *Mycoplasma* contamination using a PCR-based approach and were confirmed to be *Mycoplasma* free.

AA starvation experiments were performed as described previously^13^. In brief, custom-made starvation media were formulated according to the Gibco recipe for high-glucose DMEM, specifically omitting the AAs. The media were filtered through a 0.22-μm filter device and tested for proper pH and osmolality before use. For the respective AA-replete (+AA) treatment media, commercially available high-glucose DMEM was used (#41965039, Thermo Fisher Scientific). All treatment media were supplemented with 10% dialysed FBS. For this purpose, FBS was dialysed against 1× PBS through 3,500 MWCO dialysis tubing. For basal (+AA) conditions, the culture media were replaced with +AA treatment media 60–90 min before lysis or fixation. For AA starvation, culture media were replaced with starvation media for 1 h. For AA add-back experiments, cells were first starved as described above and then starvation media were replaced with +AA treatment media for 30 min.

### Antibodies

Antibodies against phospho-TFEB (Ser211) (#37681), TFEB (#4240), TFE3 (#14779), phospho-S6K (Thr389) (#9205), S6K (#9202), 4E-BP1 (#9452), phospho-4E-BP1 (Thr37/46) (#9459), phospho-4E-BP1 (Ser65) (9451), ULK1 (#8054), phospho-ULK1 (Ser757) (#14202), DYKDDDDK (FLAG) tag (#2368), mTOR (#2983), RagA (#4357), RagB (#8150), RagC (#9480), RagD (#4470), FLCN (#3697) and CTSD (#2284) proteins were purchased from Cell Signaling Technology. Anti-Raptor (#20984-1-AP) and anti-LARS (#21146-1-AP) antibodies were purchased from Proteintech. A monoclonal antibody recognizing human and mouse α-tubulin (#T9026) was purchased from Sigma, and the anti-HA (3F10; #11867423001) antibody was purchased from Roche. The anti-LAMP2 monoclonal antibody (DSHB Hybridoma Product H4B4) was purchased from Developmental Studies Hybridoma Bank (DSHB) and was deposited to the DSHB by August, J.T./Hildreth, J.E.K. For immunoprecipitation (IP) experiments, FLAG-tagged proteins were pulled down using anti-FLAG M2 affinity gel (#A2220, Sigma). For immunoblotting, all primary antibodies were used 1:1,000 in PBS-T, 5% BSA, except for anti-FLAG, for which 1:3,000 was used. Peroxidase-conjugated AffiniPure anti-rabbit, anti-mouse and anti-rat secondary antibodies (#711-035-152, #715-035-151 and #712-035-153, respectively; all from Jackson ImmunoResearch) were used 1:10,000 in PBS-T (1× PBS and 0.1% Tween-20), 5% powdered milk. For immunofluorescence (IF), all primary antibodies were used 1:200 in BBT solution (1× PBS, 0.1% Tween-20 and 0.1% BSA). Anti-mouse rhodamine (TRITC)-conjugated (#715-025-150, Jackson ImmunoResearch) and anti-rabbit fluorescein (FITC)-conjugated AffiniPure secondary antibodies (#711-095-152, Jackson ImmunoResearch) were used 1:100 in BBT, whereas anti-rabbit Alexa Fluor 488-conjugated (#711-545-152, Jackson ImmunoResearch) and anti-rat Alexa Fluor 647-conjugated AffiniPure secondary antibodies (#712-605-153, Jackson ImmunoResearch) were used 1:500 in BBT.

### Plasmid constructs

Expression plasmids for FLAG- and HA-tagged RagA and RagC, as well as for FLAG–Luc, were described previously^2^. The respective expression vectors for RagB and RagD were PCR amplified from pRK5–HA–GST plasmids (described in ref. ^2^) and cloned in pcDNA3-HA and pcDNA3-FLAG vectors as EcoRI/NotI inserts. For the pcDNA3-FLAG-p18 expression construct, p18 was PCR-amplified from cDNA using appropriate primers, and cloned into the EcoRI/NotI sites of pcDNA3-FLAG. The pcDNA3–HA–RagC T90N and W115R point mutants were generated by site-directed mutagenesis using appropriate primers. The RagA/B chimaeric constructs were described previously^2^. The RagDCD chimaera was generated by first constructing a RagDC plasmid (containing the RagD N-terminal tail) using a two-step overlap PCR and appropriate primers to amplify parts of RagD and RagC. The end product was cloned into the pcDNA3-HA vector as NdeI/NotI fragment. Then a GeneArt string (Thermo Fisher Scientific) was used to introduce the C-terminal RagD part as a HpaI/NotI fragment in the RagDC plasmid, generating the RagDCD expression vector.

For the cell lines stably expressing HA-tagged Rag GTPase dimers (WT, mutants and chimaeras), the respective pcDNA3-puro vectors were generated by replacing the EcoRI/ClaI pcDNA3 fragment, containing the neomycin cassette, with the StuI/BstBI fragment of the MCSV-puro plasmid (Addgene #68469, RRID:Addgene_68469; described in ref. ^61^), containing the PGK-puro cassette. The backbone and insert fragment ends were blunt before ligation.

All restriction enzymes were purchased from Fermentas/Thermo Scientific. The integrity of all constructs was verified by sequencing. Sequences of all cloning primers are provided in Supplementary Table 1.

### Generation of KO cell lines

HEK293FT knock-out (KO) cell lines were generated using the CRISPR/Cas9 system developed by the Zhang lab^62^. Double-stranded DNA oligos that encode single guide RNAs (sgRNAs) against target genes were designed using online tools. For RagB, RagC and RagD, two sgRNAs were designed per gene targeting the 5′ coding sequence or untranslated region and the 3′ coding sequence or untranslated region, respectively (Supplementary Fig. 1). RagA was efficiently knocked out using a single sgRNA. Each sgRNA was cloned into the BbsI restriction sites of the PX459 vector. The oligo sequences for all sgRNAs are provided in Supplementary Table 1.

In brief, cells were seeded in six-well plates and transfected on the following day with the respective sgRNA-expressing vectors using Effectene reagent (QIAGEN), according to the manufacturer’s instructions. Forty-eight hours post-transfection, cells were selected with 3 μg ml^−1^ puromycin (#A1113803, Thermo Fisher Scientific) for 3 days. Single-cell clones were picked by single-cell dilution, and KO clones were validated by genomic DNA PCR/sequencing (Extended Data Fig. 1) and immunoblotting using specific antibodies.

### Transient DNA transfection

Plasmid DNA transfections were performed using Effectene (QIAGEN), according to the manufacturer’s instructions.

### Stable cell line generation

For the generation of monoclonal stable lines expressing HA-tagged Rag GTPases (WT, mutants and chimaeras), HEK293FT qKO cells were transfected using the indicated Rag dimer expression vectors. Forty-eight hours post-transfection, cells were selected with 2 μg ml^−1^ puromycin for 2 days and then propagated in maintenance selection media containing the same puromycin concentration. To specifically assess the qualitative differences between the various Rag GTPase paralogues, single-cell clones that express comparable Rag levels were selected for functional characterization experiments. For the RagA versus RagB comparison, RagB expression is lower than RagA, resembling the endogenous RagA/B expression differences. Rag expression levels were validated by immunoblotting.

Despite the complications that generating monoclonal stable cell lines may introduce to a study (for example, due to clonal propagation and interclonal variability), and the fact that this is a tedious and lengthy process, this proved to be the best and only way that allows for a direct comparison between different Rag dimers and the functional characterization of their qualitative properties in the regulation of mTORC1 by AAs: while transiently overexpressing Rags could show the differences in interactions between RagC/D and other proteins in co-IP experiments, it largely masked the qualitative effects towards mTORC1 activity. This was probably due to overexpression artefacts, as Rag levels were massively higher in transiently transfected cells, compared with stable cell lines. Moreover, cells expressing such high Rag levels showed non-physiological localization patterns, with the majority of cells showing non-lysosomal Rag localization, regardless of the dimer expressed. Although polyclonal stable cell lines performed much better in maintaining the physiological regulation of mTORC1 by the Rags, they were still not appropriate for this study: because individual cells in the polyclonal population express uneven/variable Rag levels, some cells demonstrated almost undetectable Rag expression, while others had massive Rag overexpression. This led to large cell-to-cell variability, especially in microscopy studies, where we assessed mTOR or Rag localization at the single-cell level. In sum, monoclonal cell lines that express comparable Rag levels for the different Rag dimers and show low cell-to-cell variability were the only way to reliably investigate the Rag dimer code that defines the mTORC1 response to AAs.

### Immunoblotting

For immunoblotting analyses, cells were washed once in-well with serum-free DMEM, to remove FBS, and lysed in 250 µl Triton lysis buffer (50 mM Tris pH 7.5, 1% Triton X-100, 150 mM NaCl, 50 mM NaF, 2 mM Na-vanadate, 0.011 g ml^−1^ β-glycerophosphate, 1× PhosSTOP phosphatase inhibitors and 1× Complete protease inhibitors) for 10 min on ice. Samples were clarified by centrifugation (14,000*g*, 15 min, 4 °C), and supernatants were transferred to new tubes. Protein concentration was measured using the Protein Assay Dye Reagent (#5000006, Bio-Rad).

Protein samples were subjected to electrophoretic separation on SDS–PAGE and analysed by standard western blotting techniques. In brief, proteins were transferred to nitrocellulose membranes (#10600002, Amersham) and stained with 0.2% Ponceau solution (Serva) to confirm equal loading. Membranes were blocked with 5% powdered milk in PBS-T (1× PBS and 0.1% Tween-20) for 1 h at room temperature, washed three times for 10 min with PBS-T and incubated with primary antibodies (1:1,000 in PBS-T, 5% BSA) rotating overnight at 4 °C. The next day, membranes were washed three times for 10 min with PBS-T and incubated with appropriate HRP-conjugated secondary antibodies (1:10,000 in PBS-T, 5% milk) for 1 h at room temperature. Signals were detected by enhanced chemiluminescence, using the ECL Western Blotting Substrate (#W1015, Promega), or SuperSignal West Pico PLUS (#34577, Thermo Scientific) and SuperSignal West Femto Substrate (#34095, Thermo Scientific) for weaker signals. Immunoblot images were captured on film (#28906835, GE Healthcare) and quantified using the GelAnalyzer software (v19.1; www.gelanalyzer.com).

### Co-IP

For co-IP experiments, 1 × 10^6^ cells were transiently transfected with the indicated plasmids and lysed 40–48 h post-transfection in IP lysis buffer (50 mM Tris pH 7.5, 0.3% CHAPS, 150 mM NaCl, 50 mM NaF, 2 mM Na-vanadate, 0.011 g ml^−1^ β-glycerophosphate, 1× PhosSTOP phosphatase inhibitors and 1× Complete protease inhibitors). FLAG-tagged proteins were incubated with 30 μl pre-washed anti-FLAG M2 affinity gel (Sigma, #A2220) for 3 h at 4 °C and washed four times with IP wash buffer (50 mM Tris pH 7.5, 0.3% CHAPS, 150 mM NaCl and 50 mM NaF). Samples were then boiled for 6 min in 2× Laemmli sample buffer and analysed by immunoblotting using appropriate antibodies.

### LysoRag IP

To purify Rag-bound lysosomes, we developed the LysoRag IP method, a modified version of the Lyso-IP method that was previously described by the Sabatini lab^44^. This method allows for the purification of intact lysosomes, using HA-tagged Rags as bait. As a result, Rag dimers that bind to lysosomes more strongly pull down larger amounts of lysosomal material. HEK293FT qKO monoclonal cell lines, stably expressing HA-tagged RagC or RagD as dimers with RagA, were used to compare the relative affinities of RagC and RagD to lysosomes. In brief, 2 × 10^7^ cells were seeded in a 15 cm dish and allowed to settle for 24 h. On the next day, cells were washed once with ice-cold PBS and scraped in 1 ml of ice-cold PBS containing 1× PhosSTOP phosphatase inhibitors (#04906837001, Roche) and 1× Complete protease inhibitors (#11697498001, Roche). Cells were then pelleted by centrifugation (1,000*g*, 2 min, 4 °C) and resuspended in 1 ml of 1× ice-cold PBS with inhibitors. For input samples, 25 µl of the suspension was transferred in a new tube and lysed by the addition of 125 µl CHAPS lysis buffer (50 mM Tris pH 7.5, 0.3% CHAPS, 150 mM NaCl, 50 mM NaF, 2 mM Na-vanadate, 0.011 g ml^−1^ β-glycerophosphate, 1× PhosSTOP phosphatase inhibitors and 1× Complete protease inhibitors) on ice for 10 min. Lysed input samples were then cleared by centrifugation (14,000*g*, 15 min, 4 °C), and the supernatant was transferred to new tubes containing 37.5 μl of 6× Laemmli and boiled for 6 min.

For the lysosomal fractions, the remaining cell suspension was homogenized with 20 strokes in pre-chilled 2 ml hand Dounce homogenizers kept on ice. The homogenate was cleared by centrifugation (1,000*g*, 2 min, 4 °C) and incubated with 100 µl pre-washed Pierce anti-HA magnetic beads (#88837, Thermo Fisher Scientific) on a nutating mixer for 3 min at room temperature, followed by three washes with ice-cold PBS, containing phosphatase and protease inhibitors, on a DynaMag spin magnet (#12320D, Invitrogen). After the last wash, lysosomes were eluted from the beads by addition of 60 μl 2× Laemmli sample buffer and boiling for 6 min.

### IF and confocal microscopy

ΙF/confocal microscopy experiments and quantification of co-localization were performed as previously described^13^. In brief, cells were seeded on fibronectin-coated coverslips and treated as indicated in each experiment. After treatments, cells were fixed for 10 min at room temperature with 4% PFA in PBS. Samples were washed/permeabilized with PBT solution (1× PBS and 0.1% Tween-20), and blocked with BBT solution (1× PBS, 0.1% Tween-20 and 0.1% BSA). Staining was performed with the indicated primary antibodies in BBT (1:200 dilution) and then with appropriate highly cross-adsorbed secondary fluorescent antibodies (1:100 in BBT for FITC- or TRITC-conjugated antibodies; 1:500 in BBT for Alexa Fluor-conjugated antibodies). Finally, nuclei were stained with DAPI and cells mounted on slides using Fluoromount-G (#00-4958-02, Invitrogen). Images from single-channel captures are shown in greyscale. For the merged images, FITC, Alexa 647 (for anti-HA IFs), and Alexa 488 (for anti-TFE3 IFs) are shown in green, TRITC in red and DAPI in blue. Images were captured using a 40× objective lens on an SP8 Leica confocal microscope.

To quantify co-localization of mTOR or HA signal with the lysosomal marker LAMP2, the Fiji software (version 2.1.0/1.53c)^63^ was used to define regions of interest corresponding to individual cells, excluding the nucleus. Forty to 50 individual cells from approximately ten independent fields were selected per experiment for the analysis. The Coloc2 plugin was used to calculate the Manders’ co-localization coefficient, using automatic Costes thresholding^64,65^. The Manders’ co-localization coefficient yields the fraction of the signal of interest (mTOR or HA-Rag in this study) that overlaps with a second signal (in our case, lysosomes).

Subcellular localization of TFE3 was analysed by scoring cells on the basis of the signal distribution of TFE3, as shown in the example images in Fig. 2b. Signal was scored as nuclear (more TFE3 signal in the nucleus), cytoplasmic (more TFE3 signal in the cytoplasm) or intermediate (similar TFE3 signal between nucleus and cytoplasm). Approximately 50 individual cells were scored per genotype for each experiment.

### Gene expression analysis (RT–qPCR)

For gene expression analysis, RNA was isolated with TRIzol (#15596018, Thermo Fisher Scientific) and reverse transcription was performed using the RevertAid H Minus Reverse Transcriptase kit (#EP0451, Thermo Fisher Scientific). The cDNAs were diluted 1:10 in nuclease-free H_2_O and 4 µl of diluted cDNA was used per reaction, along with 5 µl of 2× Maxima SYBR Green/ROX qPCR master mix (#K0223, Thermo Fisher Scientific) and 1 µl of primer mix (2.5 µM of forward and reverse primers). For each replicate experiment, reactions were set in technical triplicates in a StepOnePlus Real-Time PCR system (Applied Biosystems) and analysed with the StepOne software (v2.2.2; Applied Biosystems). Relative gene expression levels were calculated with the 2^−^^ΔΔCt^ method. *RPL13a* expression was used for normalization as internal control.

### LysoTracker staining

For LysoTracker staining experiments, cells were seeded in fibronectin-coated coverslips and grown until they reached 80–90% confluency. Lysosomes were stained by the addition of 100 nM LysoTracker Red DND-99 (#L7528, Invitrogen) in complete medium for 1.5 h in standard culturing conditions. Cells were then fixed with 4% PFA in PBS for 10 min at room temperature, washed and permeabilized with PBT solution (1× PBS and 0.1% Tween-20), and nuclei stained with DAPI (1:2,000 in PBT) for 10 min. Coverslips were mounted on slides using Fluoromount-G (#00-4958-02, Invitrogen). Images were captured using a 40× objective lens on an SP8 Leica confocal microscope, using the Leica Application Suite X software (v3.5.7.23225). LysoTracker signal intensity was measured from 50 individual cells per genotype for each experiment using Fiji (version 2.1.0/1.53c)^63^.

### Phylogenetic analysis

Rag orthologues were identified by performing a blastp search (blastp suite) against the NCBI Reference proteins (refseq_protein; version 2021-07) database, using the AA sequences of human RRAGA (Uniprot ID: Q7L523), RRAGB (Uniprot ID: Q5VZM2-2), RRAGC (Uniprot ID: Q9HB90) and RRAGD (Uniprot ID: Q9NQL2-1) as query proteins, filtering for each of the organisms shown in Fig. 1a (*H. sapiens*, taxid: 9606; *M. mulatta*, taxid: 9544; *C. lupus*, taxid: 9615; *M. musculus*, taxid: 10090; *X. laevis*, taxid: 8355; *D. rerio*, taxid: 7955; *D. melanogaster*, taxid: 7227; *C. elegans*, taxid: 6239; *S. cerevisiae*, taxid: 4932). The expect threshold for identified proteins was set at 1 × 10^−30^; with a maximum of 100 target sequences; disabled low-complexity region filtering; using the BLOSUM62 matrix; a word size of 6; and gap existence and extension costs of 11 and 1, respectively.

### Sequence alignment and structure modelling

Structure-based sequence alignments of RagA and RagB or RagC and RagD were prepared with Clustal Omega^66^ and ESPript^67^. To generate a model of the RagA/RagC/LAMTOR complex, we superposed the crystal structure of the active RagA–Q66L–GTP/RagC–S75N–GDP heterodimer (PDBID: 6S6D)^45^ with the complex structure of LAMTOR with the dimerization domains of RagA and RagC (PDBID: 6EHR)^46^. To model the active RagB or RagD GTPases, we introduced AA substitution in the RagA or RagC GTPases (PDBID: 6S6D), respectively, in Coot^68^, followed by structure idealization using refmac5^69^. The inactive RagB/RagD dimer conformation was modelled accordingly on the basis of the cryo-EM structure of the inactive RagA/RagC dimer bound to the FLCN–FNIP2 complex (PDBID: 6ULG)^70^.

### Statistics and reproducibility

Statistical analysis and data presentation in graphs was performed using the GraphPad Prism software (v9.1.0). For all quantifications, data in the graphs are shown as mean ± standard error of the mean (s.e.m.). Normal distribution was tested using the Shapiro–Wilk or the Kolmogorov–Smirnov tests, and correction for multiple comparisons was performed using the Tukey test. Significance was calculated using unpaired two-tailed *t*-test (for pairwise comparisons, see Fig. 3b,d,f and Extended Data Figs. 3b–e,g and 4c–f) or one-way analyis of variance (for multiple comparisons, see Figs. 1c–f,h, 2b,c, 4d,e,g, 5c–f,h, 6b–e,g and 7c,e and Extended Data Figs. 2b, 6b and 7b–e,g) for normally distributed data, or the Kruskal–Wallis test for non-normally distributed data (Fig. 2e). *P* values are described in the figures and figure legends (*P < 0.05, **P < 0.01, ***P < 0.005, ****P < 0.001; NS, non-significant). Statistics source data are provided in the numerical source data table.

All findings were reproducible over multiple independent experiments, within a reasonable degree of variability between replicates. The number of replicate experiments for each assay is provided in the respective figure legends. No statistical method was used to pre-determine sample size, which was determined in accordance with standard practices in the field. No data were excluded from the analyses. The experiments were not randomized, and the investigators were not blinded to allocation during experiments and outcome assessment.

### Reporting summary

Further information on research design is available in the Nature Research Reporting Summary linked to this article.

## Online content

Any methods, additional references, Nature Research reporting summaries, source data, extended data, supplementary information, acknowledgements, peer review information; details of author contributions and competing interests; and statements of data and code availability are available at 10.1038/s41556-022-00976-y.

## Supplementary information

Supplementary Information Supplementary Figs. 1–7.

Reporting Summary

Supplementary Table 1 Oligonucleotide sequences used in the study.

## Data Availability

Uncropped immunoblots and statistics source data are provided as image source data or numerical source data files, respectively, alongside the paper. All other data supporting the findings of this study are available from the corresponding author upon reasonable request. Source data are provided with this paper.

## Source data

Source Data Fig. 1 Uncropped blots for Fig. 1b.
Source Data Fig. 1 Numerical source data.
Source Data Fig. 2 Uncropped blots for Fig. 3c.
Source Data Fig. 2 Numerical source data.
Source Data Fig. 3 Uncropped blots for Fig. 3e.
Source Data Fig. 3 Numerical source data.
Source Data Fig. 4 Uncropped blots for Fig. 4c.
Source Data Fig. 4 Numerical source data.
Source Data Fig. 5 Uncropped blots for Fig. 4f.
Source Data Fig. 5 Numerical source data.
Source Data Fig. 6 Uncropped blots for Fig. 5b.
Source Data Fig. 6 Numerical source data.
Source Data Fig. 7 Uncropped blots for Fig. 6a.
Source Data Fig. 7 Numerical source data.
Source Data Fig. 7 Uncropped blots for Fig. 7b.
Source Data Extended Data Fig. 1 Uncropped blots for Extended Data Fig. 2c.
Source Data Extended Data Fig. 2 Uncropped blots for Extended Data Fig. 2d.
Source Data Extended Data Fig. 2 Numerical source data.
Source Data Extended Data Fig. 3 Uncropped blots for Extended Data Fig. 3a.
Source Data Extended Data Fig. 3 Numerical source data.
Source Data Extended Data Fig. 4 Uncropped blots for Extended Data Fig. 4a.
Source Data Extended Data Fig. 4 Numerical source data.
Source Data Extended Data Fig. 5 Uncropped blots for Extended Data Fig. 4b.
Source Data Extended Data Fig. 6 Uncropped blots for Extended Data Fig. 6c.
Source Data Extended Data Fig. 6 Numerical source data.
Source Data Extended Data Fig. 7 Uncropped blots for Extended Data Fig. 7a.
Source Data Extended Data Fig. 7 Numerical source data.

## Code availability

No code was generated in this study.
