# Author Correction: A Rag GTPase dimer code defines the regulation of mTORC1 by amino acids

**DOI:** 10.1038/s41556-022-01073-w

**Published:** 2022-12-19

**Authors:** Peter Gollwitzer, Nina Grützmacher, Sabine Wilhelm, Daniel Kümmel, Constantinos Demetriades

**Affiliations:** 1 Max Planck Institute for Biology of Ageing (MPI-AGE), Cologne, Germany; 2 Institute of Biochemistry, University of Münster, Münster, Germany; 3 Cologne Excellence Cluster on Cellular Stress Responses in Aging-Associated Diseases (CECAD), University of Cologne, Cologne, Germany

**Keywords:** TOR signalling, Lysosomes, Nutrient signalling, Molecular modelling

Correction to: *Nature Cell Biology* 10.1038/s41556-022-00976-y, published online 12 September 2022.

The authors noticed a mistake in Supplementary Table 1 after publication. The sequences listed in rows 44–46 for oligos sgRagC_5′_rev, sgRagC_3′_for and sgRagC_3′_rev were not correct, due to copy–paste errors introduced during file preparation. The correct sequences for these oligos are now presented in rows 44–46 in the corrected Supplementary Table 1 online. All other oligo sequences were double-checked and remain accurate. The respective plasmid constructs used to knock out RagC as well as the resulting cell lines are also correct. The authors sincerely apologize for this error.

