# Supplementary material for: A Rag GTPase dimer code defines the regulation of mTORC1 by amino acids
Source: Nat Cell Biol. 2022 Sep 12;24(9):1394–406. doi: 10.1038/s41556-022-00976-y (PMC9481461; doi:10.1038/s41556-022-00976-y)
Supplement: Supplementary file 1 — Supplementary Figs. 1–7. [file 41556_2022_976_MOESM1_ESM.pdf]

nature portfolio

https://doi.org/10.1038/s41556-022-00976-y

# Supplementary information

# A Rag GTPase dimer code defines the regulation of mTORC1 by amino acids

In the format provided by the authors and unedited

**Supplementary Figures 1-7**

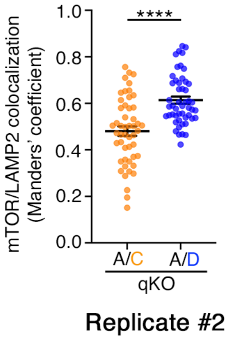


**Suppl. Fig. 1.** Replicate of experiment in Fig. 1g-h. Quantification of mTOR/LAMP2 colocalization in RagA/C and RagA/D expressing cells, showing higher lysosomal localization of mTOR in the latter. Fifty individual cells were analysed per condition. Data shown as mean ± SEM. **** p<0.001. Source numerical data are available in Source Data.

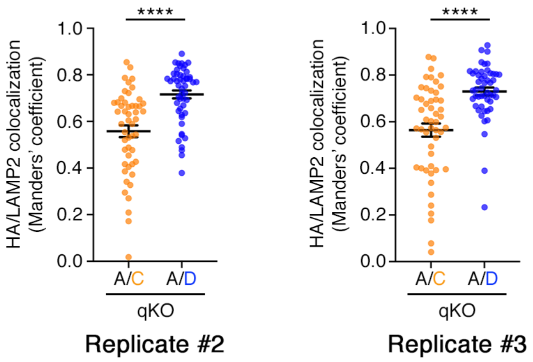


**Suppl. Fig. 2.** Replicates of experiment in Fig. 3a-b. Quantification of HA/LAMP2 colocalization in RagA/C and RagA/D expressing cells, showing higher lysosomal localization of RagD-containing dimers. The data shown in the right panel were also used in the graph in Ext. Data Fig. 5b. Fifty individual cells were analysed per condition. Data shown as mean ± SEM. **** p<0.001. Source numerical data are available in Source Data.

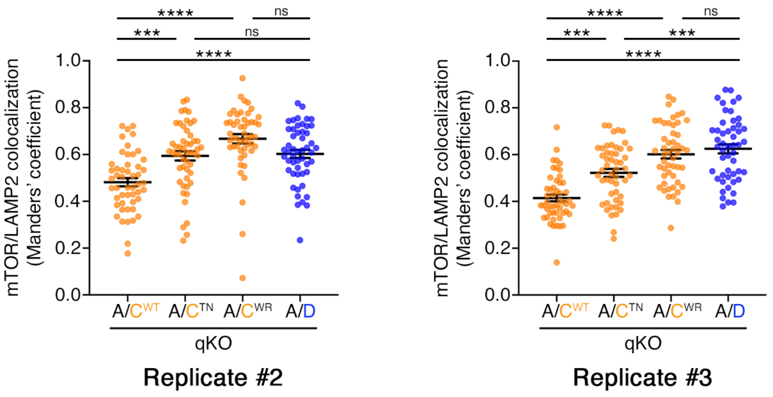


**Suppl. Fig. 3.** Replicates of experiment in Fig. 5g-h. Quantification of mTOR/LAMP2 colocalization in cells expressing wild-type RagA/C, RagA/D, or cancer-associated RagC mutants, showing higher lysosomal localization of mTOR in the RagC mutants ($C^{TN}$, $C^{WR}$), compared to WT RagC ($C^{WT}$). Fifty individual cells were analysed per condition. Data shown as mean ± SEM. *** p<0.005, **** p<0.001. Source numerical data are available in Source Data.

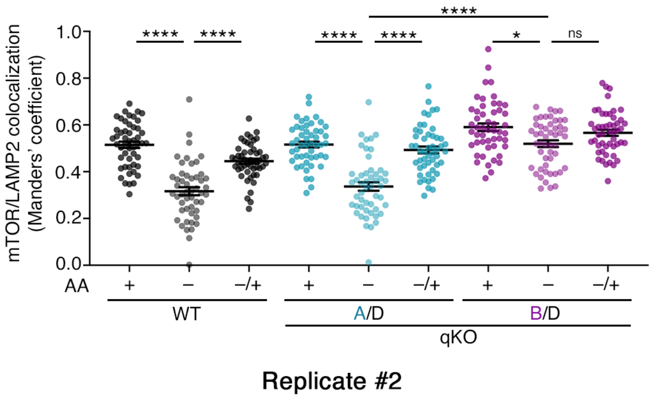


**Suppl. Fig. 4.** Replicate of experiment in Fig. 6f-g. Quantification of mTOR/LAMP2 colocalization in WT, RagA/D, and RagB/D expressing cells, showing compromised AA starvation response and incomplete delocalization of mTOR from lysosomes in the latter. Fifty individual cells were analysed per condition. Data shown as mean ± SEM. * p<0.05, **** p<0.001. Source numerical data are available in Source Data.

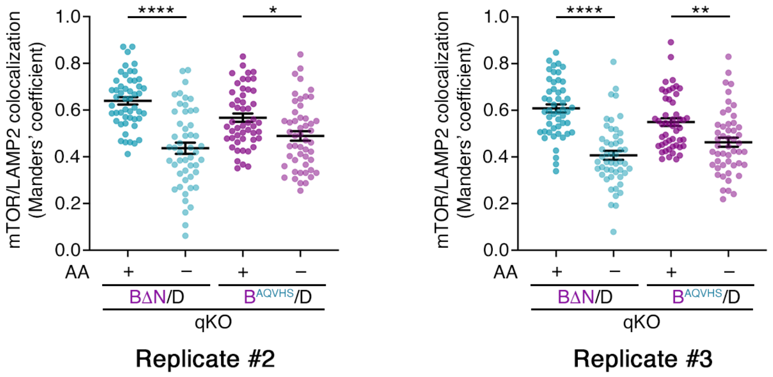


**Suppl. Fig. 5.** Replicates of experiment in Fig. 7d-e. Quantification of mTOR/LAMP2 colocalization in WT, RagA/C and RagB/D expressing cells, showing compromised AA starvation response and incomplete delocalization of mTOR from lysosomes in the latter. Fifty individual cells were analysed per condition. Data shown as mean ± SEM. * p<0.05, ** p<0.01, **** p<0.001. Source numerical data are available in Source Data.

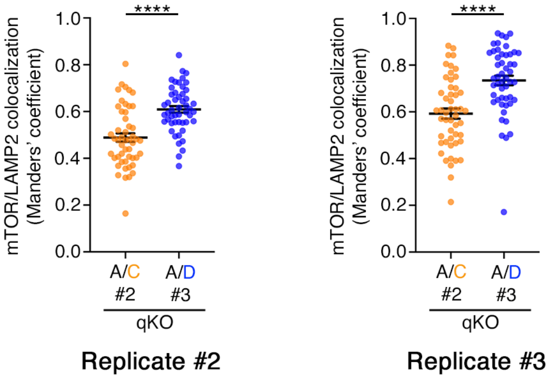


**Suppl. Fig. 6.** Replicates of experiment in Ext. Data Fig. 3f-g. Quantification of mTOR/LAMP2 colocalization in RagA/C and RagA/D expressing cells (additional clones #2), showing higher lysosomal localization of mTOR in the latter. Fifty individual cells were analysed per condition. Data shown as mean ± SEM. **** p<0.001. Source numerical data are available in Source Data.

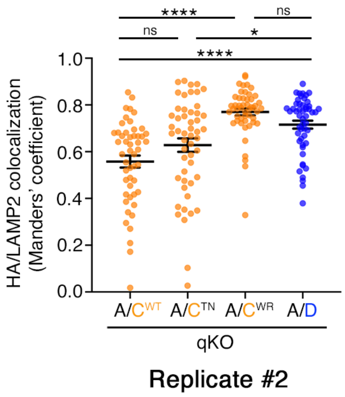


**Suppl. Fig. 7.** Replicate of experiment in Ext. Data Fig. 6a-b. Quantification of HA/LAMP2 colocalization in cells expressing wild-type RagA/C, RagA/D, or cancer-associated RagC mutants, showing higher lysosomal localization of the RagC mutants ($C^{TN}$, $C^{WR}$), compared to WT RagC ($C^{WT}$). Fifty individual cells were analysed per condition. Data shown as mean ± SEM. * $p<0.05$, ** $p<0.01$, *** $p<0.005$, **** $p<0.001$. Source numerical data are available in Source Data.
