# Supplementary material for: A Rag GTPase dimer code defines the regulation of mTORC1 by amino acids
Source: Nat Cell Biol. 2022 Sep 12;24(9):1394–406. doi: 10.1038/s41556-022-00976-y (PMC9481461; doi:10.1038/s41556-022-00976-y)
Supplement: Supplementary file 2 — Reporting Summary [file 41556_2022_976_MOESM2_ESM.pdf]

nature portfolio

Corresponding author(s): Dr Constantinos Demetriades

Last updated by author(s): Jun 24, 2022

Reporting Summary

Nature Portfolio wishes to improve the reproducibility of the work that we publish. This form provides structure for consistency and transparency in reporting. For further information on Nature Portfolio policies, see our Editorial Policies and the Editorial Policy Checklist.

Statistics

For all statistical analyses, confirm that the following items are present in the figure legend, table legend, main text, or Methods section.

n/a	Confirmed	
☐	☒	The exact sample size (*n*) for each experimental group/condition, given as a discrete number and unit of measurement
☐	☒	A statement on whether measurements were taken from distinct samples or whether the same sample was measured repeatedly
☐	☒	The statistical test(s) used AND whether they are one- or two-sided<br>*Only common tests should be described solely by name; describe more complex techniques in the Methods section.*
☒	☐	A description of all covariates tested
☐	☒	A description of any assumptions or corrections, such as tests of normality and adjustment for multiple comparisons
☐	☒	A full description of the statistical parameters including central tendency (e.g. means) or other basic estimates (e.g. regression coefficient) AND variation (e.g. standard deviation) or associated estimates of uncertainty (e.g. confidence intervals)
☐	☒	For null hypothesis testing, the test statistic (e.g. *F*, *t*, *r*) with confidence intervals, effect sizes, degrees of freedom and *P* value noted<br>*Give P values as exact values whenever suitable.*
☒	☐	For Bayesian analysis, information on the choice of priors and Markov chain Monte Carlo settings
☒	☐	For hierarchical and complex designs, identification of the appropriate level for tests and full reporting of outcomes
☒	☐	Estimates of effect sizes (e.g. Cohen's *d*, Pearson's *r*), indicating how they were calculated

Our web collection on statistics for biologists contains articles on many of the points above.

Software and code

Policy information about availability of computer code

Data collection

No code for data collection was generated in this study.

Commercial software packages from other developers used in this study are:

Confocal microscopy: Leica Application Suite X 3.5.7.23225; https://www.leica-microsystems.com/products/microscope-software/p/leica-las-x-ls/
qPCR data acquisition: StepOne software, Applied Biosystems, Version 2.2.2; https://www.thermofisher.com/de/de/home/technical-resources/software-downloads/StepOne-and-StepOnePlus-Real-Time-PCR-System.html

Data analysis

No code for data analysis was generated in this study.

Commercial software packages from other developers used in this study are:

Statistical analysis and graph preparation: GraphPad Prism 9.1.0 (216); https://www.graphpad.com/
Quantification of colocalization: Fiji Version 2.1.0 / 1.53c; https://imagej.net/software/fiji/
Quantification of immunoblots: GelAnalyzer 19.1; http://www.gelanalyzer.com/
Sequence alignment: Clustal Omega (https://www.ebi.ac.uk/Tools/msa/clustalo/); ESPript 3.0 (https://espript.ibcp.fr/ESPript/ESPript/)
Modelling of Rag structure: WinCoot 0.8.9 and Refmac5; https://www.ccp4.ac.uk/download/

For manuscripts utilizing custom algorithms or software that are central to the research but not yet described in published literature, software must be made available to editors and reviewers. We strongly encourage code deposition in a community repository (e.g. GitHub). See the Nature Portfolio guidelines for submitting code & software for further information.

March 2021

Data

Policy information about availability of data

All manuscripts must include a data availability statement. This statement should provide the following information, where applicable:

- Accession codes, unique identifiers, or web links for publicly available datasets
- A description of any restrictions on data availability
- For clinical datasets or third party data, please ensure that the statement adheres to our policy

Uncropped immunoblots and statistics source data are provided as Image Source Data or Numerical Source Data files, respectively, alongside the paper. All other data supporting the findings of this study are available from the corresponding author upon reasonable request. Structure modelling was performed using published structures as described in the 'Structure alignment and Structure modelling' part of the Methods section.

Field-specific reporting

Please select the one below that is the best fit for your research. If you are not sure, read the appropriate sections before making your selection.

☒ Life sciences ☐ Behavioural & social sciences ☐ Ecological, evolutionary & environmental sciences

For a reference copy of the document with all sections, see nature.com/documents/nr-reporting-summary-flat.pdf

Life sciences study design

All studies must disclose on these points even when the disclosure is negative.

Sample size	No statistical methods were used for sample size determination, which was determined in accordance with standard practices in the field and based on our long-standing experience in this type of experimental approaches (e.g., PMIDs 26868506, 33497611, 33974911).<br>For colocalization analysis, 40-50 cells from multiple independent fields were quantified per condition per experiment.<br>For qPCR gene expression analysis, data from 6-7 independent experiments are shown, with 3 technical replicates per experiment.
Data exclusions	No data were excluded from the analyses.
Replication	All findings were reproducible over multiple independent experiments, within a reasonable degree of variability between replicates. The numbers of replicate experiments are provided in the respective figure legends.
Randomization	Sample randomization was not performed for the experiments described in this study, as the order of analysis does not influence the experimental outcomes.
Blinding	No blinding was included in the data collection or analysis, as the method of quantification over multiple replicates and individual cells (for microscopy experiments) ensures unbiased processing.

Reporting for specific materials, systems and methods

We require information from authors about some types of materials, experimental systems and methods used in many studies. Here, indicate whether each material, system or method listed is relevant to your study. If you are not sure if a list item applies to your research, read the appropriate section before selecting a response.

Materials & experimental systems

n/a	Involved in the study	
☐	☒	Antibodies
☐	☒	Eukaryotic cell lines
☒	☐	Palaeontology and archaeology
☒	☐	Animals and other organisms
☒	☐	Human research participants
☒	☐	Clinical data
☒	☐	Dual use research of concern

Methods

n/a	Involved in the study	
☒	☐	ChIP-seq
☒	☐	Flow cytometry
☒	☐	MRI-based neuroimaging

Antibodies

Antibodies used	Primary antibodies<br>Rabbit, monoclonal, anti-phospho-TFEB (Ser211), clone E9S8N, #37681, Cell Signaling Technology, Lot #2<br>Rabbit, polyclonal, ant-TFEB, #4240, Cell Signaling Technology, Lot #3<br>Rabbit, polyclonal, anti-TFE3, #14779, Cell Signaling Technology, Lot #1<br>Rabbit, monoclonal, anti-phospho-S6K (Thr389), clone 108D2, #9234, Cell Signaling Technology, Lot #12

Rabbit, polyclonal, anti-S6K, #9202, Cell Signaling Technology, Lot #20
Rabbit, polyclonal, anti-4E-BP1, #9452, Cell Signaling Technology, Lot #12
Rabbit, polyclonal, ant-phospho-4E-BP1 (Thr37/46), #9459, Cell Signaling Technology, Lot #10
Rabbit, polyclonal, ant-phospho-4E-BP1 (Ser65), #9451, Cell Signaling Technology, Lot #16
Rabbit, monoclonal, anti-ULK1, clone D8H5, #8054, Cell Signaling Technology, Lot #7
Rabbit, monoclonal, anti-phospho-ULK1 (Ser757), clone D7O6U, #14202, Cell Signaling Technology, Lot #5
Rabbit, polyclonal, anti-DYKDDDDK Tag, #2368, Cell Signaling Technology, Lot #12
Rabbit, monoclonal, anti-mTOR, clone 7C10, #2983, Cell Signaling Technology, Lot #19
Rabbit, monoclonal, anti-RagA, clone D8B5, #4357, Cell Signaling Technology, Lot #3
Rabbit, monoclonal, anti-RagB, clone D18F3, #8150, Cell Signaling Technology, Lot #1
Rabbit, monoclonal, anti-RagC, clone D8H5, #9480, Cell Signaling Technology, Lot #2
Rabbit, polyclonal, anti-RagD, #4470, Cell Signaling Technology, Lot #2
Rabbit, monoclonal, anti-FLCN, clone D14G9, #3697, Cell Signaling Technology, Lot #3
Rabbit, polyclonal, anti-Cathepsin D, #2284, Cell Signaling Technology, Lot #2
Rabbit, polyclonal, anti-Raptor, #20984-1-AP, Proteintech
Rabbit, polyclonal, anti-LARS, #21146-1-AP, Proteintech
Mouse, monoclonal, anti-alpha-tubulin, clone DM1A, #T9026, Sigma
Rat, monoclonal, anti-HA, clone 3F10, #11867423001, Roche
Mouse, monoclonal, anti-LAMP2, clone H4B4, Developmental Studies Hybridoma Bank

Secondary antibodies
Peroxidase-conjugated AffiniPure donkey anti-rabbit IgG (H+L), polyclonal, #711-035-152, Jackson ImmunoResearch
Peroxidase-conjugated AffiniPure donkey anti-mouse IgG (H+L), polyclonal, #715-035-151, Jackson ImmunoResearch
Peroxidase-conjugated AffiniPure donkey anti-rat IgG (H+L), polyclonal, #712-035-153, Jackson ImmunoResearch
Rhodamine (TRITC)-conjugated AffiniPure donkey anti-mouse IgG (H+L), polyclonal, #715-025-150, Jackson ImmunoResearch
Fluorescein (FITC)-conjugated AffiniPure donkey anti-rabbit IgG (H+L), polyclonal, #711-095-152, Jackson ImmunoResearch
Alexa Fluor 488-conjugated AffiniPure donkey anti-rabbit IgG (H+L), polyclonal, #711-545-152, Jackson ImmunoResearch
Alexa Fluor 647-conjugated AffiniPure donkey anti-rat IgG (H+L) polyclonal, #712-605-153, Jackson ImmunoResearch

Validation

Specificity of phospho-antibodies extensively verified in this study and in the context of other projects in the Demetriades lab, using inhibitors for the respective kinases or starvation media.
Specificity of total protein antibodies extensively verified in this study and in the context of other projects in the Demetriades lab, using knock-out cell lines, or knock-down and overexpression experiments.

Additional information for all antibodies used in this study can be found in the manufacturer's website for each product:

anti-phospho-TFEB (Ser211), #37681, Cell Signaling Technology, validated for Western Blotting, relevant citations can be found on the manufacturer's website (https://www.cellsignal.com/products/primary-antibodies/phospho-tfeb-ser211-e9s8n-rabbit-mab/37681)

anti-TFEB, #4240, Cell Signaling Technology, validated for Western Blotting and Immunoprecipitation, relevant citations can be found on the manufacturer's website (https://www.cellsignal.com/products/primary-antibodies/tfeb-antibody/4240)

anti-TFE3, #14779, Cell Signaling Technology, validated for Western Blotting, relevant citations can be found on the manufacturer's website (https://www.cellsignal.com/products/primary-antibodies/tfe3-antibody/14779)

anti-phospho-S6K (Thr389), #9234, Cell Signaling Technology, validated for Western Blotting, relevant citations can be found on the manufacturer's website (https://www.cellsignal.com/products/primary-antibodies/phospho-p70-s6-kinase-thr389-108d2-rabbit-mab/9234)

anti-S6K, #9202, Cell Signaling Technology, validated for Western Blotting and Immunoprecipitation, relevant citations can be found on the manufacturer's website (https://www.cellsignal.com/products/primary-antibodies/p70-s6-kinase-antibody/9202)

anti-4E-BP1, #9452, Cell Signaling Technology, validated for Western Blotting, relevant citations can be found on the manufacture's website (https://www.cellsignal.com/products/primary-antibodies/4e-bp1-antibody/9452)

anti-phospho-4E-BP1 (Thr37/46), #9459, Cell Signaling Technology, validated for Western Blotting, relevant citations can be found on the manufacture's website (https://www.cellsignal.com/products/primary-antibodies/phospho-4e-bp1-thr37-46-antibody/9459)

anti-phospho-4E-BP1 (Ser65), #9451, Cell Signaling Technology, validated for Western Blotting, relevant citations can be found on the manufacture's website (https://www.cellsignal.com/products/primary-antibodies/phospho-4e-bp1-ser65-antibody/9451)

anti-ULK1, #8054, Cell Signaling Technology, validated for Western Blotting, relevant citations can be found on the manufacture's website
(https://www.cellsignal.com/products/primary-antibodies/ulk1-d8h5-rabbit-mab/8054)

anti-phospho-ULK1 (Ser757), #14202, Cell Signaling Technology, validated for Western Blotting, relevant citations can be found on the manufacture's website
(https://www.cellsignal.com/products/primary-antibodies/phospho-ulk1-ser757-d7o6u-rabbit-mab/14202)

anti-DYKDDDDK Tag, #2368, Cell Signaling Technology, validated for Western Blotting, Immunoprecipitation and Flow Cytometry, relevant citations can be found on the manufacturer's website (https://www.cellsignal.com/products/primary-antibodies/dykddddk-tag-antibody-binds-to-same-epitope-as-sigma-s-anti-flag-m2-antibody/2368)

anti-mTOR, #2983, Cell Signaling Technology, validated for Western Blotting, Immunohistochemistry, Immunofluorescence and Flow Cytometry, relevant citations can be found on the manufacturer's website (https://www.cellsignal.com/products/primary-antibodies/

mtor-7c10-rabbit-mab/2983)

anti-RagA, #4357, Cell Signaling Technology, validated for Western Blotting and Immunoprecipitation, relevant citations can be found on the manufacturer's website (https://www.cellsignal.com/products/primary-antibodies/raga-d8b5-rabbit-mab/4357)

anti-RagB, #8150, Cell Signaling Technology, validated for Western Blotting and Immunoprecipitation, relevant citations can be found on the manufacturer's website (https://www.cellsignal.com/products/primary-antibodies/ragb-d18f3-rabbit-mab/8150)

anti-RagC, #9480, Cell Signaling Technology, validated for Western Blotting, Immunoprecipitation, Immunofluorescence and Flow Cytometry, relevant citations can be found on the manufacturer's website (https://www.cellsignal.com/products/primary-antibodies/ragc-d8h5-rabbit-mab/9480)

anti-RagD, #4470, Cell Signaling Technology, validated for Western Blotting, relevant citations can be found on the manufacturer's website (https://www.cellsignal.com/products/primary-antibodies/ragd-antibody/4470)

anti-FLCN, #3697, Cell Signaling Technology, validated for Western Blotting and Immunoprecipitation, relevant citations can be found on the manufacturer's website (https://www.cellsignal.com/products/primary-antibodies/flcn-d14g9-rabbit-mab/3697)

anti-Cathepsin D, #2284, Cell Signaling Technology, validated for Western Blotting and Immunohistochemistry, relevant citations can be found on the manufacturer's website (https://www.cellsignal.com/products/primary-antibodies/cathepsin-d-antibody/2284)

anti-Raptor, #20984-1-AP, proteintech, validated for Western Blotting, Immunoprecipitation, Immunohistochemistry and Immunofluorescence, relevant citations can be found on the manufacturer's website (https://www.ptglab.com/products/RPTOR-Antibody-20984-1-AP.htm#tested-applications)

anti-LARS, #21146-1-AP, proteintech, validated for Western Blotting, Immunoprecipitation, Immunohistochemistry and Immunofluorescence, relevant citations can be found on the manufacturer's website (https://www.ptglab.com/products/LARS-Antibody-21146-1-AP.htm)

anti-alpha-tubulin, #T9026, Sigma, validated for Western Blotting and Immunofluorescence, relevant citations can be found on the manufacturer's website (https://www.sigmaaldrich.com/DE/en/product/sigma/t9026)

anti-HA, #11867423001, Roche, validated for Western Blotting, Dot Blots, ELISA, Immunocytochemistry and Immunoprecipitation, relevant citations can be found on the manufacturer's website (https://www.sigmaaldrich.com/DE/en/product/roche/roahaha)

anti-LAMP2, clone H4B4, Developmental Studies Hybridoma Bank, validated for Western Blotting, FACS, Function Blocking, Immunofluorescence, Immunohistochemistry and Immunoprecipitation, relevant citations can be found on the manufacturer's website (https://dshb.biology.uiowa.edu/H4B4)

Eukaryotic cell lines

Policy information about cell lines

Cell line source(s)	The parental HEK293FT cells were purchased from Invitrogen before the initiation of the project.
Authentication	The identity of the parental HEK293FT cells was validated by the Multiplex human Cell Line Authentication test (Multiplexion GmbH), which uses a single nucleotide polymorphism (SNP) typing approach, and was performed as described at www.multiplexion.de.
Mycoplasma contamination	All cell lines were regularly tested for Mycoplasma contamination, using a PCR-based approach and were confirmed to be Mycoplasma-free.
Commonly misidentified lines (See ICLAC register)	No commonly misidentified lines were used in this study.
