# Supplementary figures and images for: A Rag GTPase dimer code defines the regulation of mTORC1 by amino acids
Source: Nat Cell Biol. 2022 Sep 12;24(9):1394–406. doi: 10.1038/s41556-022-00976-y (PMC9481461; doi:10.1038/s41556-022-00976-y)

## Uncropped blots for Fig. 1b

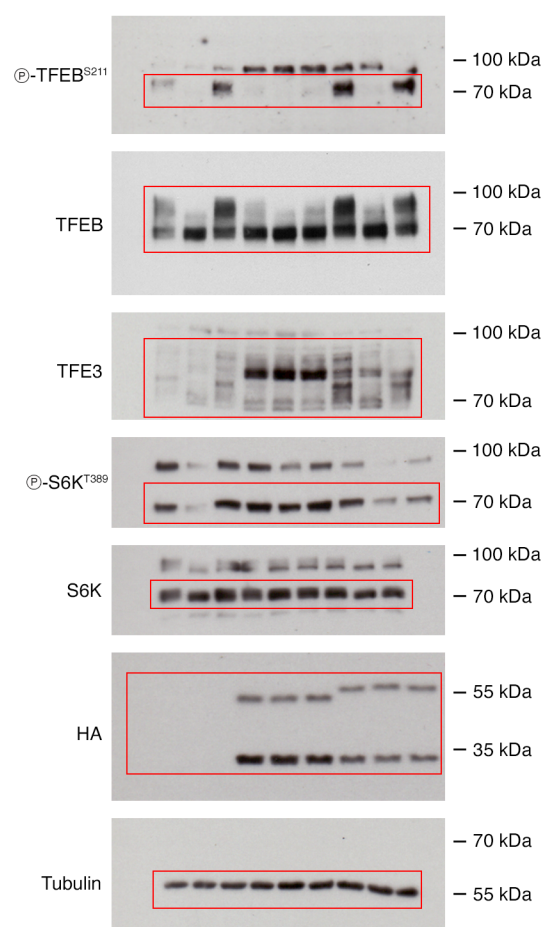

Supplement: Source Data Fig. 1 — Uncropped blots for Fig. 1b. [file 41556_2022_976_MOESM4_ESM.pdf]

### Uncropped blots for Fig. 3c

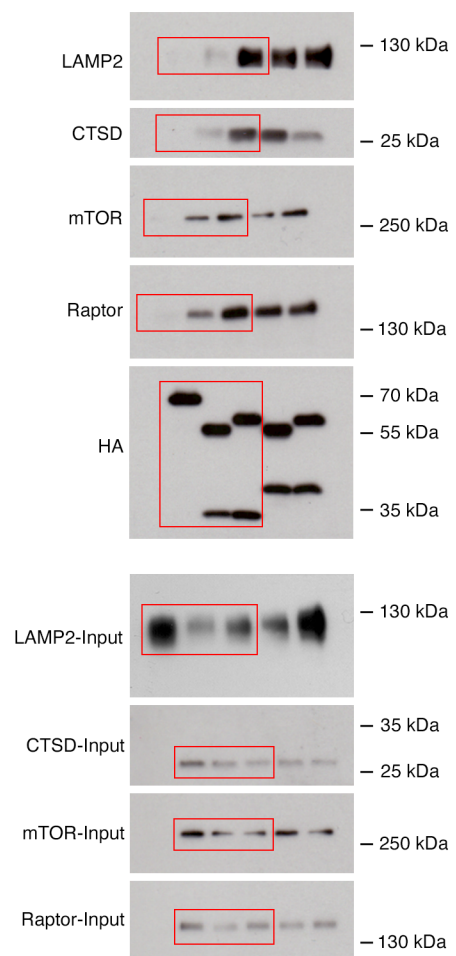

Supplement: Source Data Fig. 2 — Uncropped blots for Fig. 3c. [file 41556_2022_976_MOESM6_ESM.pdf]

### Uncropped blots for Fig. 3e

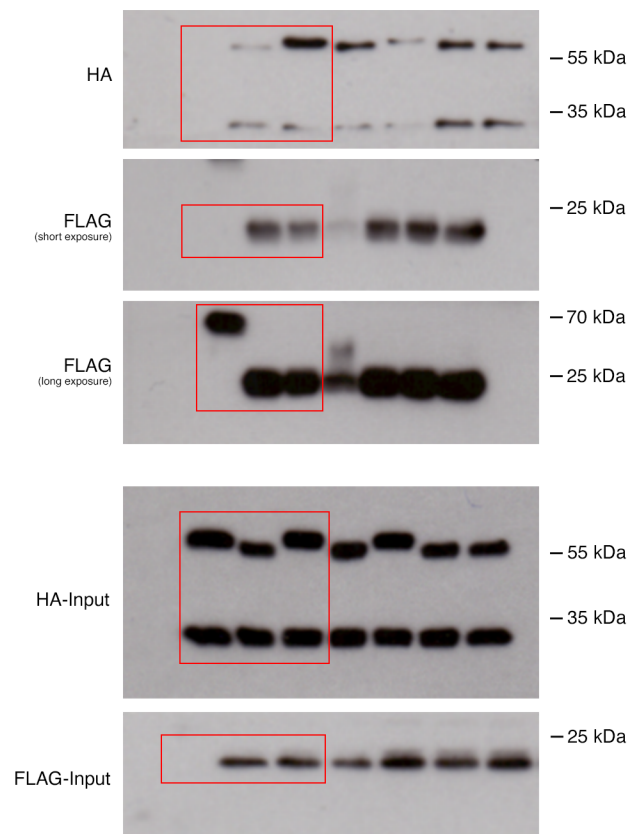

Supplement: Source Data Fig. 3 — Uncropped blots for Fig. 3e. [file 41556_2022_976_MOESM8_ESM.pdf]

# Uncropped blots for Fig. 4c

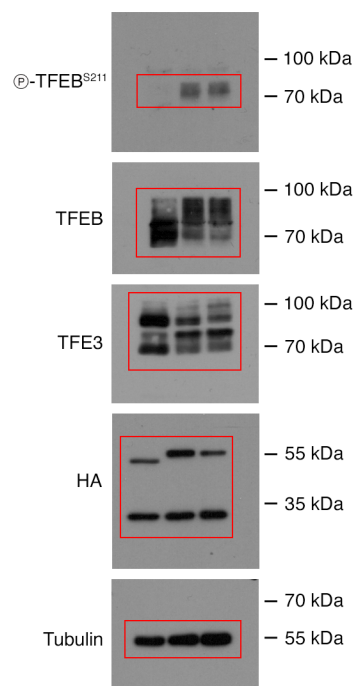

Supplement: Source Data Fig. 4 — Uncropped blots for Fig. 4c. [file 41556_2022_976_MOESM10_ESM.pdf]

# Uncropped blots for Fig. 4f

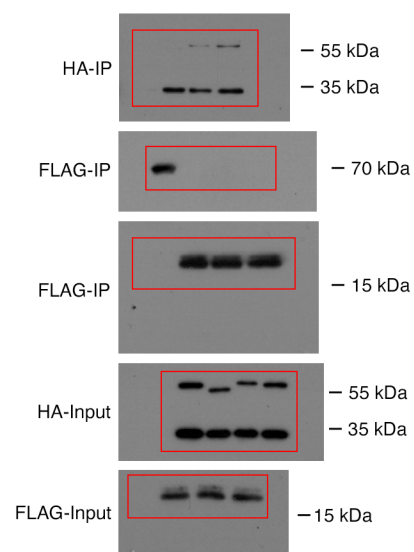

Supplement: Source Data Fig. 5 — Uncropped blots for Fig. 4f. [file 41556_2022_976_MOESM12_ESM.pdf]

## Uncropped blots for Fig. 5b

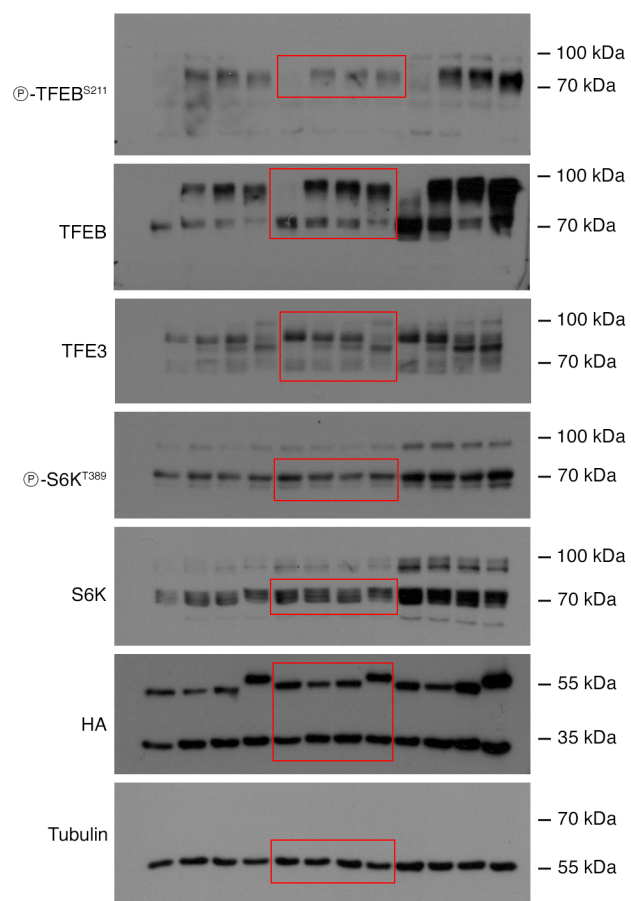

Supplement: Source Data Fig. 6 — Uncropped blots for Fig. 5b. [file 41556_2022_976_MOESM14_ESM.pdf]

## Uncropped blots for Fig. 6a

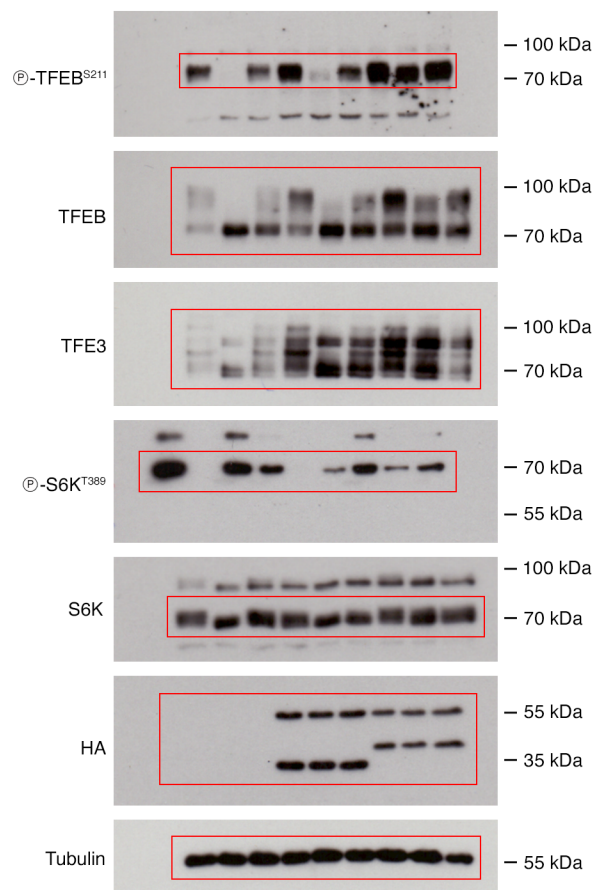

Supplement: Source Data Fig. 7 — Uncropped blots for Fig. 6a. [file 41556_2022_976_MOESM16_ESM.pdf]

## Uncropped blots for Fig. 7b

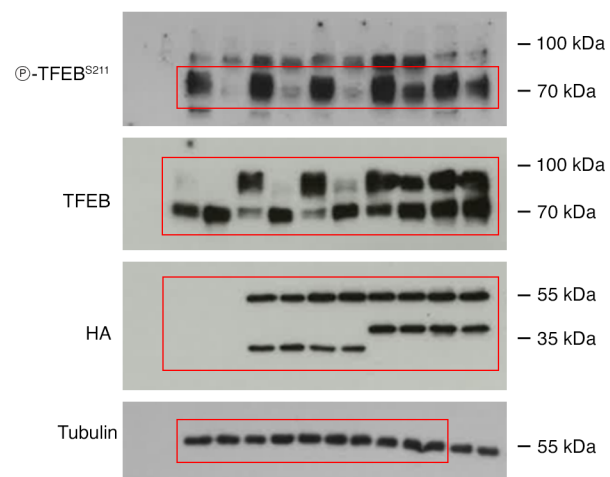

Supplement: Source Data Fig. 7 — Uncropped blots for Fig. 7b. [file 41556_2022_976_MOESM18_ESM.pdf]

Uncropped blots for Extended Data Fig. 2c

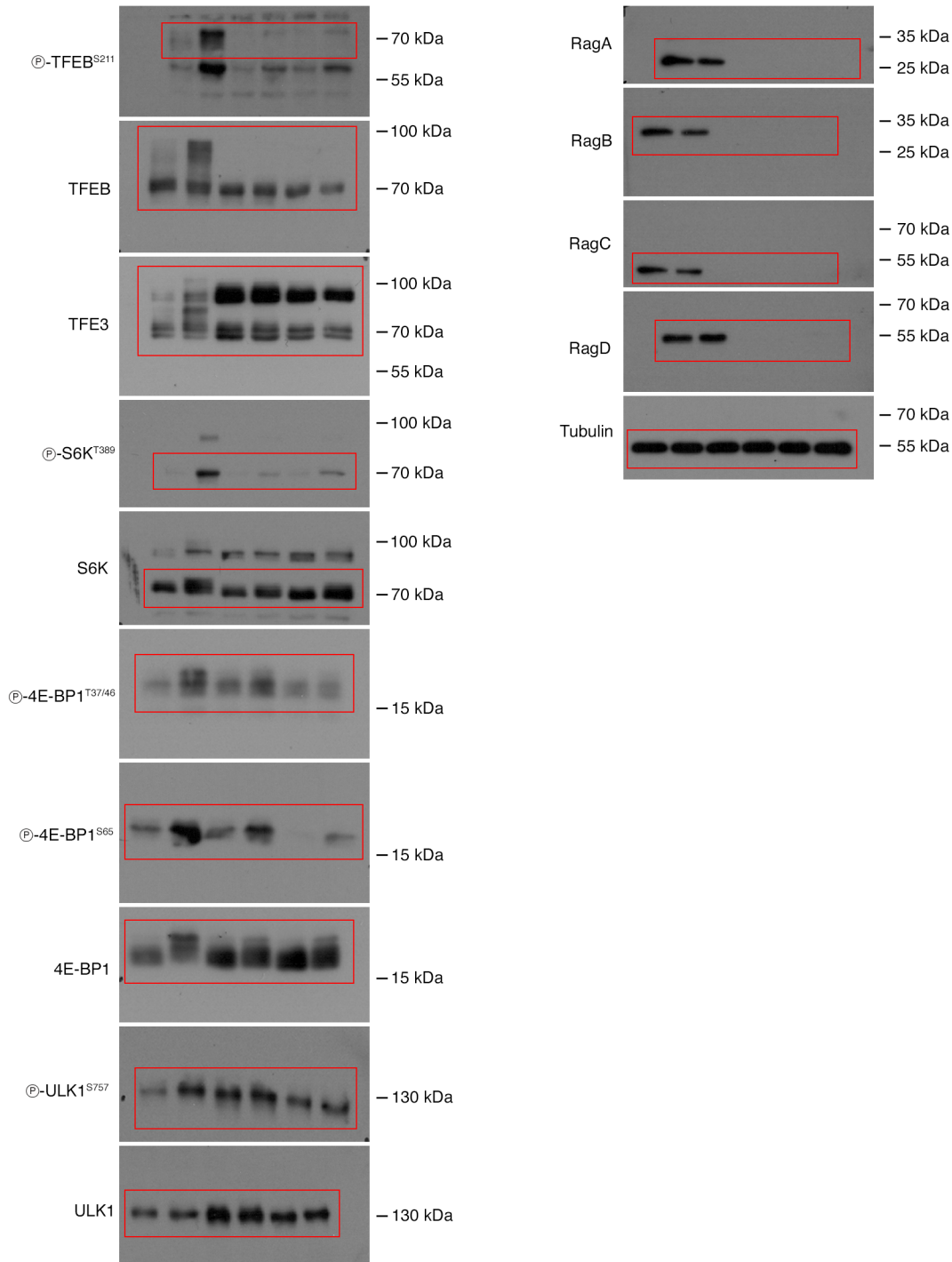

Supplement: Source Data Extended Data Fig. 1 — Uncropped blots for Extended Data Fig. 2c. [file 41556_2022_976_MOESM19_ESM.pdf]

## Uncropped blots for Extended Data Fig. 2d

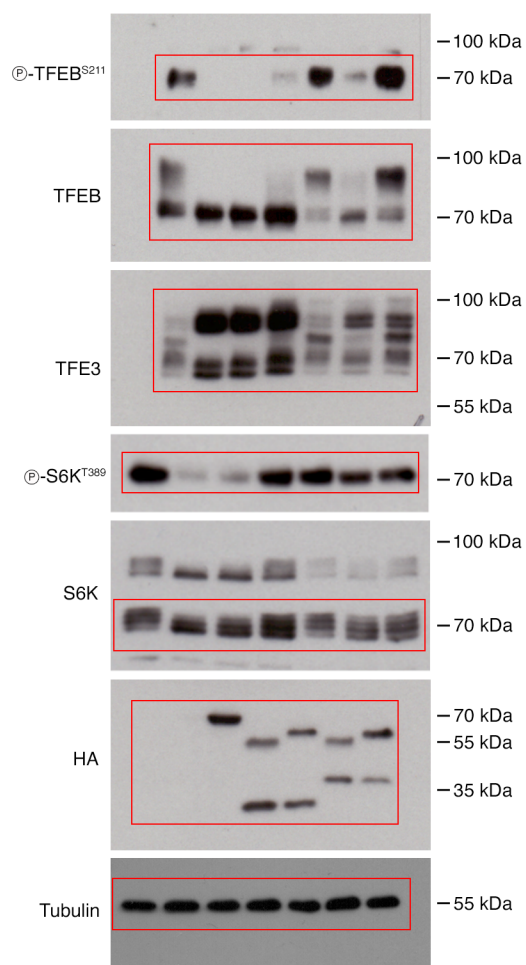

Supplement: Source Data Extended Data Fig. 2 — Uncropped blots for Extended Data Fig. 2d. [file 41556_2022_976_MOESM20_ESM.pdf]

# Uncropped blots for Extended Data Fig. 3a

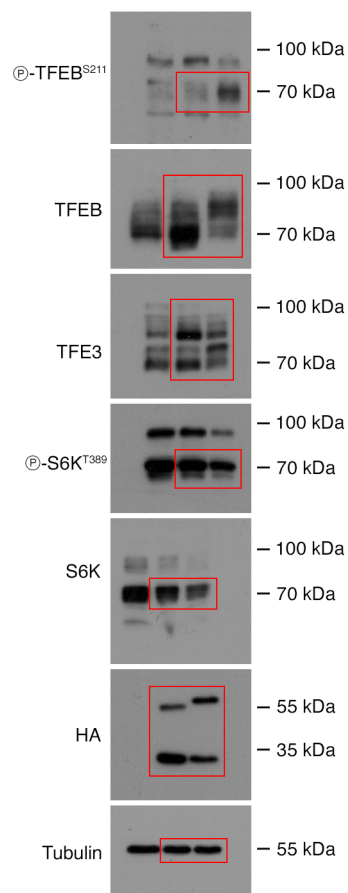

Supplement: Source Data Extended Data Fig. 3 — Uncropped blots for Extended Data Fig. 3a. [file 41556_2022_976_MOESM22_ESM.pdf]

## Uncropped blots for Extended Data Fig. 4a

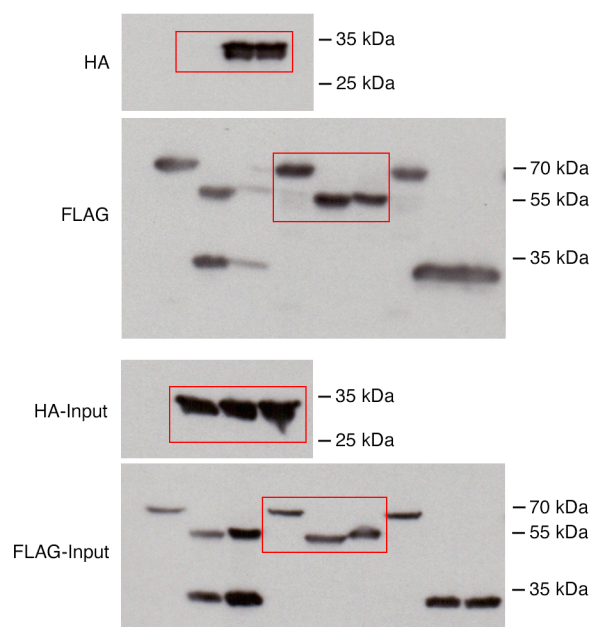

Supplement: Source Data Extended Data Fig. 4 — Uncropped blots for Extended Data Fig. 4a. [file 41556_2022_976_MOESM24_ESM.pdf]

## Uncropped blots for Extended Data Fig. 4b

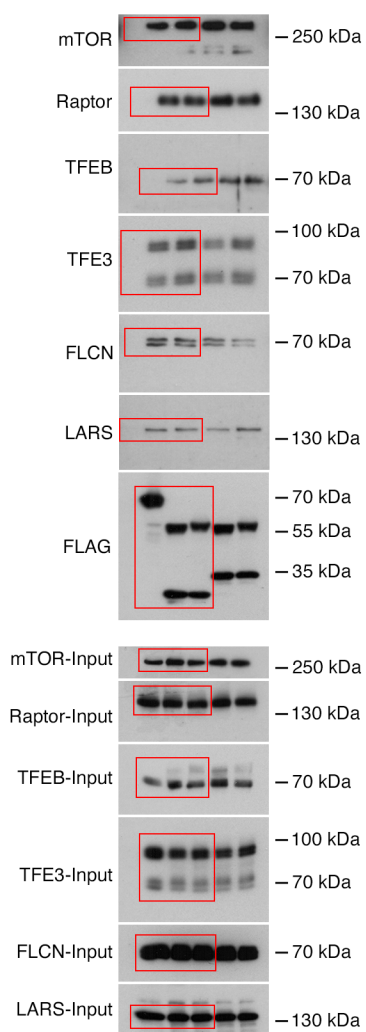

Supplement: Source Data Extended Data Fig. 5 — Uncropped blots for Extended Data Fig. 4b. [file 41556_2022_976_MOESM26_ESM.pdf]

# Uncropped blots for Extended Data Fig. 6c

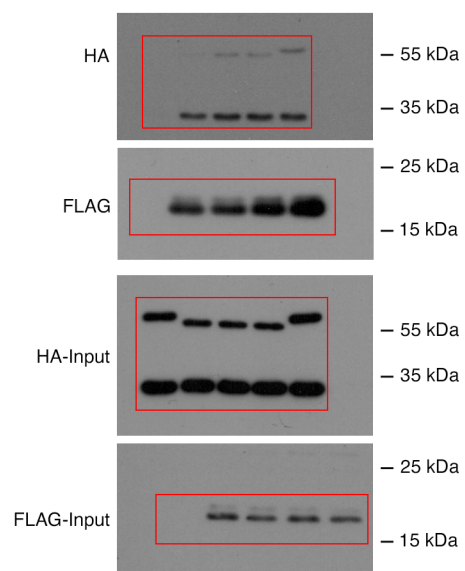

Supplement: Source Data Extended Data Fig. 6 — Uncropped blots for Extended Data Fig. 6c. [file 41556_2022_976_MOESM27_ESM.pdf]

## Uncropped blots for Extended Data Fig. 7a

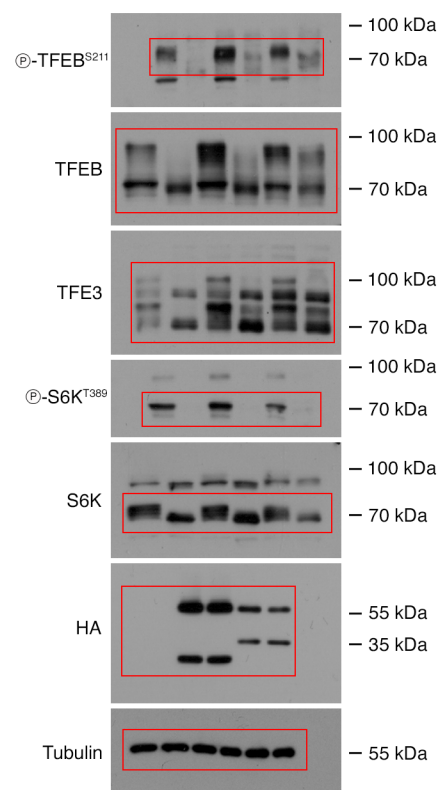

Supplement: Source Data Extended Data Fig. 7 — Uncropped blots for Extended Data Fig. 7a. [file 41556_2022_976_MOESM29_ESM.pdf]
